# Designing MOF–Thermogel Nanocomposites for Differential Multidrug Release in Combination Cancer Therapy

**DOI:** 10.1021/acsanm.5c03527

**Published:** 2025-08-24

**Authors:** Wenyi Zeng, Tristan T. Y. Tan, Qianyu Lin, Wei Wei Loh, Yan Hui Lee, Michael R. Reithofer, Xian Jun Loh, Jia Min Chin, Jason Y. C. Lim

**Affiliations:** † Institute of Materials Research and Engineering (IMRE), Agency for Science, Technology and Research (A*STAR), 2 Fusionopolis Way, Innovis #08-03, Singapore 138634, Republic of Singapore; ‡ Institute of Inorganic Chemistry, Faculty of Chemistry, 27258 University of Vienna, Währinger Str. 42, 1090 Vienna, Austria; § Department of Materials Science and Engineering, National University of Singapore (NUS), 9 Engineering Drive, Singapore 117576, Republic of Singapore; ∥ Institute of Functional Materials and Catalysis, Faculty of Chemistry, University of Vienna, Währinger Str. 42, 1090 Vienna, Austria

**Keywords:** metal−organic framework, hydrogel composites, controlled drug release, synergistic chemotherapy, localized tumor treatment

## Abstract

Combination chemotherapy is a leading strategy for advanced cancer treatment, bringing about improved therapeutic responses compared with single-drug chemotherapy. However, achieving the required sequence of drug delivery needed for optimal therapeutic benefits via a single-drug delivery system remains highly challenging, often involving systems of considerable complexities. Herein, we report the design of composites comprising nanoscale metal–organic frameworks (MOFs) and temperature-responsive hydrogels (thermogels) as versatile, modular, yet simple-to-formulate platforms for controlled, localized release of combination chemotherapeutics, which can be used for solid tumor treatment. First, the encapsulation behavior, drug–host interactions, and *in vitro* release kinetics of four chemotherapeutic drugs—gemcitabine (GEM), 5-fluorouracil (5-FU), doxorubicin (DOX), and paclitaxel (PTX)—from nanoscale MOF carriers and the bulk gel phase were elucidated. Based on these differences, we designed dual- and even triple-drug formulations that could achieve sustained drug release over 10–18 days, with different rates of drug release that mimic clinically relevant sequential dosage. In all cases, MOF–thermogel multidrug formulations were highly injectable when chilled, potentially allowing minimally invasive and site-specific administration of multidrug cocktails to targeted tumor sites. Our findings establish MOF–thermogel nanocomposites as a highly customizable platform for tailoring multidrug release kinetics, relative rates, sequence, and release duration to meet different therapeutic demands for solid tumor chemotherapy and related applications.

## Graphical abstract

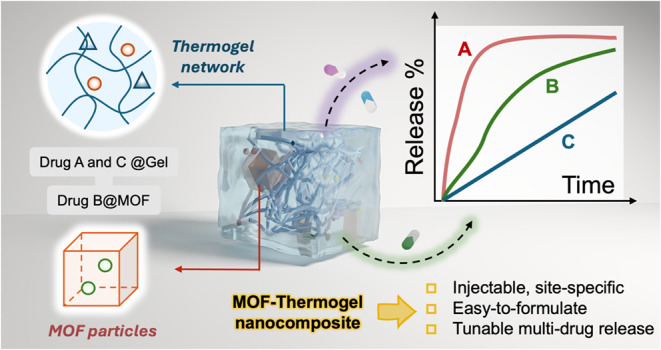

## Introduction

Combination chemotherapy, which involves the use of two or more therapeutic agents, has emerged as a powerful strategy in modern cancer treatment.^1^ Compared with traditional monotherapy, combined regimens offer significant advantages by targeting multiple molecular pathways or cellular mechanisms. This approach has been demonstrated to enhance treatment efficacy, reduce individual drug dosages, minimize adverse side effects, suppress drug resistance, and improve overall survival rates across various cancer types.^1^ For instance, doxorubicin (DOX), a commonly used anthracycline in cancer therapy, exerts its antitumor effects through DNA intercalation and triggering apoptosis.^2^ However, its clinical use is often limited by dose-dependent toxicities, including acute myelosuppression, mucositis, and cumulative cardiac toxicity.^2^ To address these limitations, combination therapy strategies have been investigated. Gemcitabine (GEM) is a nucleoside analogue and metabolic inhibitor with broad-spectrum antitumor activity.^3^ It is known for its mild hematologic toxicity, good patient tolerance, and low risk of inducing multidrug resistance.^4^ In patients with advanced breast cancer, coadministration of DOX with GEM has shown to improve the objective response rate without a significant rise in high-grade toxicities.^5^ In addition, in clinical studies of nonsmall-cell lung cancer (NSCLC), GEM paired with paclitaxel (PTX) has achieved efficacy due to the complementary mechanisms of action and partially nonoverlapping toxicity profiles.^6^ Paclitaxel, a microtubule-stabilizing agent, promotes tubulin polymerization and disrupts the microtubule dynamics, resulting in mitotic arrest and cell apoptosis.^7,8^ In this drug pair, PTX can enhance the antitumor efficacy of GEM by upregulating deoxycytidine kinase (dCK), a key enzyme involved in GEM activation, which helps to increase the intracellular concentration of GEM and its active metabolites. The use of GEM+PTX combination in clinical trials has led to significantly improved overall survival rate in patients with advanced NSCLC.^6^

In addition to drug selection, the sequence of administration plays a critical role in optimizing the therapeutic efficacy. *In vitro* studies using the triple-negative breast cancer cell line MDA-MB-231 have demonstrated clear schedule-dependent cytotoxicity.^9^ Administering GEM prior to DOX enhances treatment synergy, which is associated with increased caspase activity and apoptosis. This sequence-dependent effect allows for the use of lower drug dosages, potentially reducing the risk of neutropenia and other adverse effects. Reports on lung cancer cell lines (e.g., A549 and H520) have also revealed that GEM exposure prior to PTX showed superior tumor suppression compared to the reverse order.^10^ This synergism is attributed to GEM-induced S-phase cell cycle arrest along with enhanced tubulin acetylation and microtubule polymerization, which together sensitize tumor cells to subsequent PTX treatment.^11^

Despite the promising potential of combination therapies, effective codelivery of multiple chemotherapeutic agents remains a major clinical challenge. Currently, combination regimens are commonly administrated via separated intravenous injections or infusions, which can result in nonlocalized drug distribution, burst release, and systemic cell toxicity.^12^ Additionally, differences in the physicochemical properties of individual drugs, such as solubility, stability, and clearance rates, may bring out complicated and unpredictable pharmacokinetics as well as pharmacodynamics. Patients are also subjected to multicycle, weekly treatments that often span several months, imposing considerable socioeconomic burdens on both healthcare systems and patients.

To overcome these challenges, the development of advanced drug delivery systems has become a major focus in cancer nanomedicine. These platforms are designed to coencapsulate and release multiple agents in a spatially- and temporally controlled manner, thereby enhancing treatment efficacy, reducing dosing frequency, and minimizing systemic toxicity.^13^ Conventional nanocarrier systems including liposomes, micelles, and nanoparticles have been extensively studied to improve drug solubility, bioavailability, prolong circulation time, and achieve targeted delivery.^13^ Nevertheless, these systems face several limitations that hinder their use in combination therapy. While liposomes are biocompatible and capable of encapsulating both hydrophilic and hydrophobic agents, they are disadvantaged by low drug loading capacity, short circulation half-life, poor physical stability, and are prone to rapid clearance from the body.^14^ Micelles, typically used for delivering hydrophobic drugs, exhibit thermodynamic instability and tend to disassemble upon dilution in the bloodstream, leading to premature drug release.^15^ Other strategies, such as usage of multistimuli-responsive layer-by-layer microcapsules^16^ and specific enzyme-triggered release of drugs covalently conjugated to carrier molecules,^17^ can achieve spatiotemporal drug release for combination therapy. However, these are challenging and costly to synthesize, potentially limiting their scalability for clinical translation.

In recent years, the potential of nanoscale metal–organic frameworks (MOFs) in advanced biomedical applications, such as drug delivery, is gradually being realized.^18,19^ Other than their large internal surface areas that allow high drug loadings, MOFs offer exceptional bottom-up designability for tuning lattice dimensions, pore size, drug–MOF interactions, drug release kinetics, and release strategies.^20,21^ While MOFs can protect drugs from premature degradation, their potential instability under physiological conditions and tendency for burst release of drugs can limit their standalone use.^22^ To mitigate these limitations, hybrid systems integrating MOFs with other functional materials, such as hydrogels, have emerged as a promising strategy.^23^ Among different types of hydrogels, supramolecular thermogels are particularly suited for sustained drug delivery: their spontaneous temperature-triggered sol-to-gel phase transition enables them to be easily administered via injection when chilled, forming a persistent drug release depot upon subsequent warming to physiological temperature (Figure 1).^24^ The amphiphilic thermogelling copolymers are hydrated and exist as unimers at low temperatures. Upon warming, their hydrophobic segments dehydrate, causing spontaneous formation of micelles. When further warmed above their gelation temperature (designed to be lower than the physiological temperature), the micelles aggregate into a three-dimensional supramolecular network that entraps water without requiring any additional cross-linkers, hence bringing about the macroscopic sol–gel phase transition. Their macroscopic properties (i.e., gel stiffness, viscosity, gelation temperature) can be tuned at a molecular level by varying the ratio and identity of hydrophobic to hydrophilic components in the amphiphilic copolymers. Integrating MOFs into thermogels has been shown to improve the MOFs’ biocompatibility and prolong the release duration of drug payloads from MOFs.^25^ Additionally, their exceedingly simple preparation, achievable by mixing appropriate combinations of drug-loaded MOFs with chilled aqueous solutions of thermogelling copolymers, can bring about simultaneous release of different drugs at varying rates. The modularity of MOF–thermogel composites, enabled by independently tuning the release behavior of both MOF and thermogel components, makes this platform especially suited for achieving the control of sequence, dosage, and duration of drug delivery required for anticancer combination therapy.

**Figure 1:**
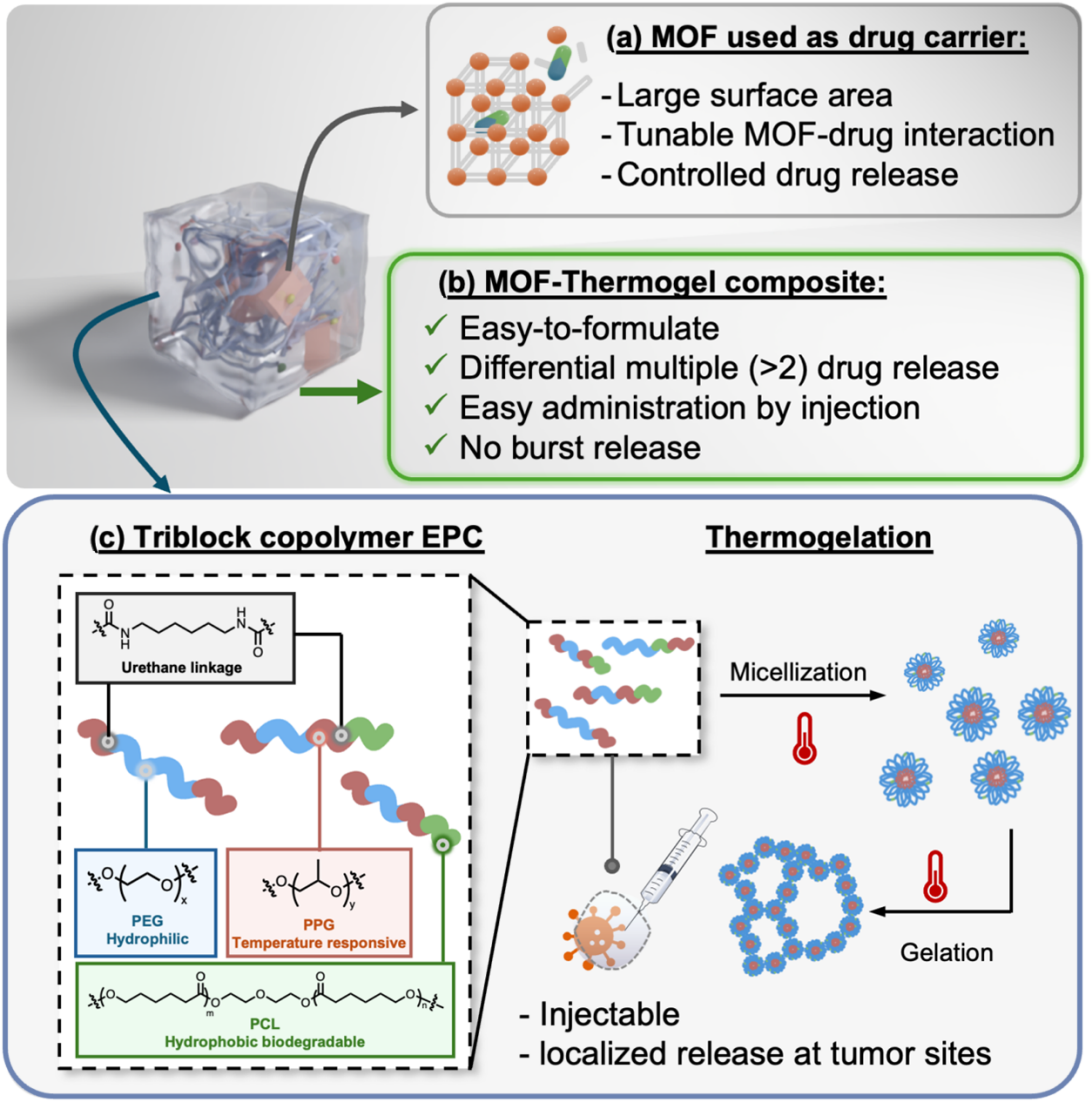
(a) Key advantages of MOF as drug delivery carriers. (b) Benefits of the MOF–thermogel composite for drug delivery. (c) Left: Chemical structure of the EPC random multiblock copolymer, consisting of PEG–PPG–PCL segments linked via urethane bonds. Right: Schematic illustration of the thermogelation process showing the sol-to-gel phase transition triggered by temperature elevation.

Herein, we demonstrate the ease of designing MOF–thermogel nanocomposites for achieving sequence-specific differential drug release relevant to three distinct clinically relevant chemotherapeutic drug combinations for cancer treatment, allowing differential release of up to three drugs simultaneously. The composites were formulated simply by mixing biocompatible MOFs in appropriate combinations with a thermogel composed of an amphiphilic polyurethane triblock copolymer (named EPC), comprising poly­(ethylene glycol) (PEG), poly­(propylene glycol) (PPG), and poly­(caprolactone) (PCL) (Figure 1c). Each component was loaded with selected combinations of chemotherapeutic drugs based on an understanding of their drug release behavior. In all combinations, the thermogels retain their characteristic sol-to-gel transition below physiological temperature, allowing the composites to be easily administered via injection. This enables localized delivery of the chemotherapeutic agents to the tumor site, preventing systemic toxicity and undesirable side effects from intravenous and oral administrations of these drugs.^26,27^ Additionally, site-specific delivery also enables therapeutically relevant dosage of drugs to reach the targeted tumor site, which is especially challenging for many hydrophobic chemotherapeutic drugs (e.g., paclitaxel) that exhibit low solubility, resulting in only small amounts of drug reaching the tumor.^28^ By spatially compartmentalizing drug encapsulation, our study showcases the potential of MOF–thermogel nanocomposites as a simple, customizable, and modular differential drug release platform for cancer treatment and other therapeutic needs.

## Experimental Methods

### Preparation and Characterization of the EPC Gel

Poly­(ethylene glycol) (PEG, 80 g), poly­(propylene glycol) (PPG, 20 g), and poly­(ε-caprolactone)-diol (PCL, 1 g) were weighed out in a 1 L round-bottomed flask. The polymers were dissolved in anhydrous toluene (120 mL) at 60 °C and twice dried by azeotropic distillation via rotary evaporation, followed by heating under high vacuum at 110 °C for 1 h. After drying, anhydrous toluene (450 mL) was added to the reaction mixture, and the reaction was heated with stirring at 110 °C until a clear solution formed. Dibutyltin dilaurate (DBTL, 0.1 mL) was added portionwise as a catalyst, followed by portionwise addition of hexamethylene diisocyanate (HMDI, 8.19 mL) to achieve a 1:1 OH:NCO molar ratio. Thereafter, the reaction was stirred under an argon atmosphere for 1.5 h, resulting in a clear, viscous solution.

The reaction was quenched by adding absolute ethanol (20 mL) and stirred for an additional 30 min before cooling to room temperature. The crude polymer was precipitated by pouring it into diethyl ether (4500 mL) at room temperature. The obtained product was collected by decantation as a white solid and dried overnight under air.

To further purify the polymer, the precipitate was dissolved in isopropyl alcohol at a ratio of 1 g per 40 mL. The resulting solution was transferred into 3.5 kDa dialysis membranes (200 mL per tube) and dialyzed against deionized water (5 L per tube) for 4 days, with water replacement every 5–6 h. After dialysis, the sample was frozen completely and freeze-dried for 3 days to obtain the final product (92% yield).

### Synthesis of MOFs (UiO-66, UiO-66-NH_2_, MOF-808, and PTX@ZIF-8)

#### UiO-66

Zirconium­(IV) chloride (360 mg, 1.5 mmol) and terephthalic acid (384 mg, 2.3 mmol) were added to 60 mL of DMF and the reaction mixture was sonicated until fully dissolved. After the addition of deionized water (400 μL), the mixture was heated in an oven at 110 °C for 24 h. After the reaction mixture was cooled to room temperature, the solid was isolated by centrifugation and washed with DMF and MeOH three times, followed by drying in an oven at 60 °C. The white powder was then activated at 200 °C for 2 h.

#### UiO-66-NH_2_

Zirconium­(IV) chloride (116.2 mg, 0.5 mmol) and 2-aminoterephthalic acid (90.5 mg, 0.5 mmol) were added to 15 mL of DMF. Acetic acid (1.43 mL, 25 mmol, 50 equiv) was added to the reaction mixture. Then, the reaction mixture was heated at 120 °C overnight. After the reaction mixture was cooled to room temperature, the off-white solid was separated via centrifugation and washed twice with DMF and twice with methanol. After the last wash, the mixture in methanol was transferred to a reaction flask and refluxed at 90 °C for 10 days. Then, the solid product was separated via centrifugation and dried in an oven at 60 °C overnight.

#### MOF-808

MOF-808 was synthesized as previously reported.^29^ First, zirconium­(IV) chloride (2 g, 8.6 mmol) was mixed with acetic acid (3 mL) and isopropanol (5 mL), followed by heating at 120 °C for 1 h. The solid was then separated by centrifugation, washed with acetone twice, and dried under vacuum. The synthesized Zr_6_ oxoclusters (0.6 g) were dissolved in a mixture of formic acid (3 mL) and DI H_2_O (5 mL), forming a colorless solution. Trimesic acid (150 mg, 0.7 mmol) was added to the reaction mixture and stirred overnight. The resulting white solid was separated via centrifugation, washed with DI H_2_O and ethanol and dried in the oven at 60 °C overnight.

#### PTX@ZIF-8

Paclitaxel (50 mg) and zinc nitrate hexahydrate were dissolved in methanol (10 mL) under sonication for 10 min. Then, a solution of 2-methylimidazole (330 mg) in 10 mL of DI H_2_O was slowly added to the PTX/Zn solution under stirring. Then, the reaction mixture was kept at room temperature for 2 days. Afterward, the solid part was separated via centrifugation, washed further three times with DI H_2_O, twice with methanol, and dried in the oven at 60 °C overnight.

### Drug Encapsulation in MOFs

Drug-loaded MOFs were synthesized by the impregnation of as-synthesized MOF particles with a drug solution under stirring at room temperature. The encapsulated drug concentration was quantified by high-performance liquid chromatography (HPLC) (see Section S4).

#### For Gemcitabine (GEM) Encapsulation

100 mg portions of MOF samples UiO-66 and UiO-66-NH_2_ were mixed with 30 mg of GEM in 5 mL of HEPES buffer solution (25 mM, pH 7). The suspension was stirred at room temperature for 3 days. Then, the MOF was recovered by centrifugation and washed three times with DI H_2_O to remove the excess drug on the surface. The drug-loaded MOFs, denoted as GEM@UiO-66 and GEM@UiO-66-NH_2_, were further dried at 60 °C.

#### For 5-Fluorouracil (5-FU) Encapsulation

100 mg of MOF samples UiO-66 and UiO-66-NH_2_ were mixed with 30 mg of 5-FU in 5 mL of HEPES buffer solution (25 mM, pH 7). The suspension was stirred at room temperature for 3 days. Then, the MOF was recovered by centrifugation and washed three times with DI H_2_O to remove the excess drug on the surface. The drug-loaded MOFs, denoted as 5-FU@UiO-66 and 5-FU@UiO-66-NH_2_, were further dried at 60 °C.

#### For Doxorubicin (DOX) Encapsulation

50 mg portion of MOF-808 was mixed with 10 mg of DOX in 5 mL of HEPES buffer solution (25 mM, pH 7). The suspension was stirred at room temperature for 3 days. Then, the MOF was recovered by centrifugation and washed three times with DI H_2_O to remove the excess drug on the surface. The drug-loaded MOF, denoted as DOX@MOF-808, was further dried at 60 °C.

### Fabrication of MOF/Gel Composite for the *In Vitro* Release Study

All drug-loaded EPC hydrogels were prepared in triplicates in 2.5 mL Eppendorf tubes, and 1 mL of HEPES buffer (25 mM, pH 7) was added above the gel layer. Concentrations of gels (wt/wt %) were determined by $(\frac{\mathrm{mass}\;\mathrm{of}\;\mathrm{polymer}}{\mathrm{mass}\;\mathrm{of}\;\mathrm{polymer}\;+\;\mathrm{buffer}}\times \;100)\%$ .

#### GEM@UiO-66/EPC

70 mg portion of EPC gel was weighed and dissolved in 200 μL of DI H_2_O at 4 °C overnight. 200 μL of GEM@UiO-66 stock suspension in HEPES buffer (3 mg/mL) was added to EPC gel solution. The mixture was equilibrated at 4 °C overnight. Afterward, the gel mixture was warmed at 37 °C to form a gel.

#### GEM@UiO-66/DOX@EPC

70 mg of EPC gel was weighed and dissolved in 100 μL of DI H_2_O at 4 °C overnight. 200 μL of GEM@UiO-66 stock suspension in HEPES buffer (3 mg/mL) and 100 μL of stock solution of DOX (0.5 mg/mL in HEPES buffer) were added to EPC gel solution. The mixture was equilibrated at 4 °C overnight. Afterward, the gel mixture was warmed at 37 °C to form a gel.

#### GEM@UiO-66/PTX@EPC

70 mg of EPC gel was weighed and dissolved in 190 μL of DI H_2_O at 4 °C overnight. 200 μL of GEM@UiO-66 stock suspension in HEPES buffer (3 mg/mL) and 10 μL of stock solution of PTX (5 mg/mL in ethanol) were added to EPC gel solution. The mixture was equilibrated at 4 °C overnight. Afterward, the gel mixture was warmed at 37 °C to form a gel.

#### GEM@UiO-66/5-FU+DOX@EPC

70 mg of EPC gel was weighed and dissolved in 100 μL of DI H_2_O at 4 °C overnight. 200 μL of GEM@UiO-66 stock suspension in HEPES buffer (3 mg/mL), 50 μL of stock solution of DOX (1 mg/mL in HEPES buffer), and 50 μL of stock solution of 5-FU (1 mg/mL in HEPES buffer) were added to the EPC gel solution. The mixture was equilibrated at 4 °C overnight. Afterward, the gel mixture was warmed at 37 °C to form a gel.

The triplicated samples for each formulation were incubated in a 37 °C oven under continuous shaking. After a certain time, 0.8 mL of an aliquot of supernatant was extracted from the sample tube, followed by addition of fresh 0.8 mL of 25 mM HEPES buffer (pH 7). In the case where PTX was used, the buffer system was replaced by 25 mM HEPES buffer (pH 7) with 0.03 w/v% sodium dodecyl sulfate (SDS).

## Results and Discussion

### Synthesis and Characterizations of Drug-Loaded MOFs

Four clinically approved chemotherapeutic agents—5-fluorouracil (5-FU), gemcitabine (GEM), doxorubicin (DOX) and paclitaxel (PTX)—with varying hydrophobicity, molecular sizes, and distinct mechanisms of action in cancer treatment were selected in this study to evaluate their encapsulation and release behavior in the hybrid MOF–thermogel composite (Figure 2a). To identify a suitable MOF carrier for the drugs, we screened four representatives MOFs-UiO-66, UiO-66-NH_2_, MOF-808, and ZIF-8, all of which are known for their low cytotoxicity,^30^ with different pore and window sizes (Figure 2b). For drug encapsulation, the as-synthesized MOFs were activated and dispersed in the respective drug solution at room temperature for 3 days. The integrity of the MOF samples was characterized by powder X-ray diffraction (PXRD), showing no significant loss of crystallinity after drug encapsulation (see Figure S1). Scanning electron microscopy (SEM) images indicated that the drug-loaded MOFs are relatively monodisperse with an average particle size below 1 μm (Figure S2). For each drug@MOF combination, the loading efficacy was quantified by high-performance liquid chromatography (HPLC) (see Section S4) and calculated using the following equation

$$loading\;in\;wt\;\%=\frac{mass\;of\;loaded\;drug}{total\;mass\;of\;MOF}\;\times \;100\%$$

**Figure 2:**
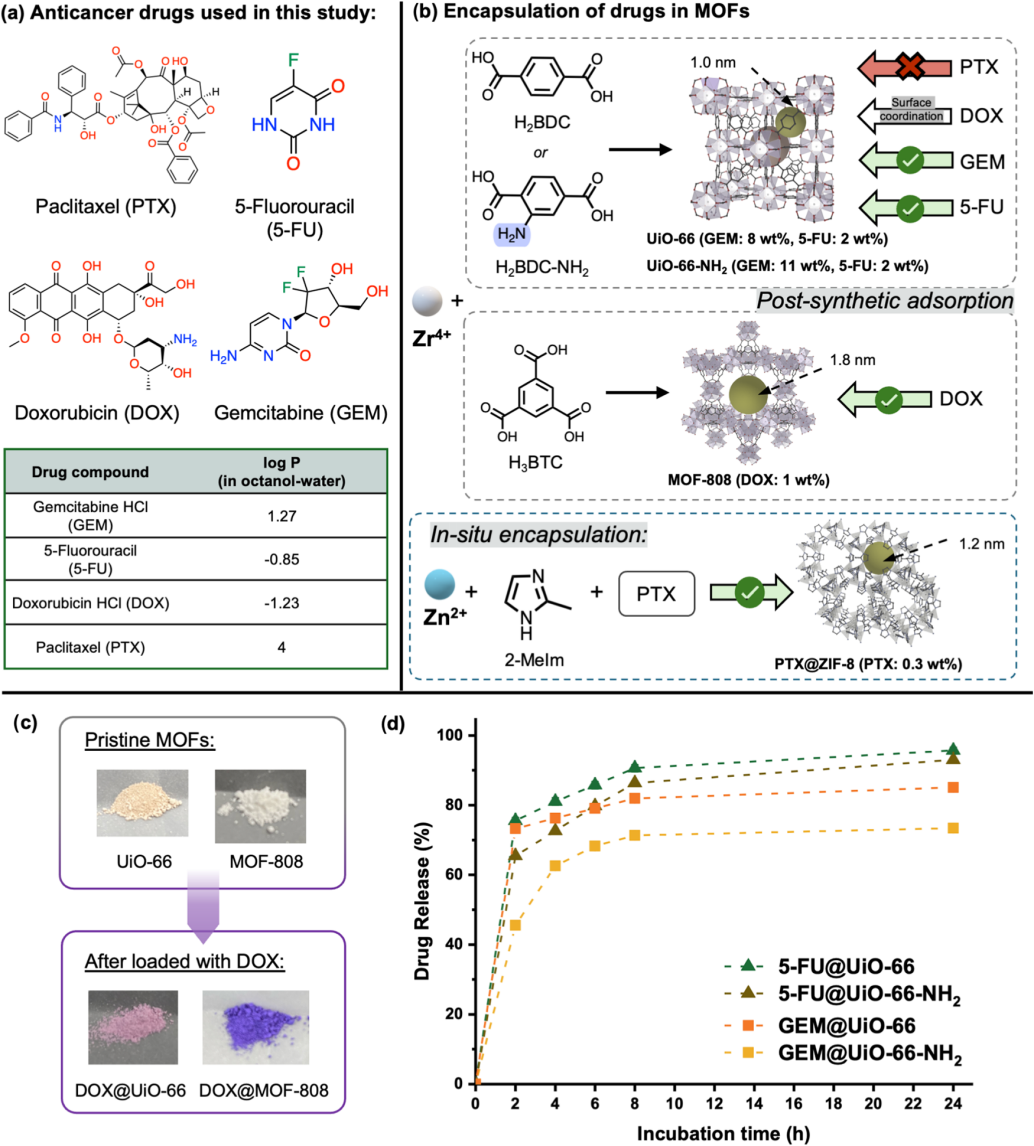
(a) Molecular structures and log *P* ^38–41^ of the anticancer drugs used in this study. (b) Selected MOF and drug combinations for encapsulation. Pore sizes are indicated by yellow spheres, and drug loadings are shown in brackets. The crystal structures were generated from published CIFs.^42–45^ (c) Photographs of MOF samples (UiO-66, DOX@UiO-66, MOF-808, and DOX@MOF-808), showing visible color changes resulting from DOX encapsulation. (d) Cumulative release profiles of GEM and 5-FU from UiO-66 and UiO-66-NH_2_, respectively, within 24 h at 37 °C in 25 mM HEPES buffer (pH 7).

For small-molecule drugs such as 5-FU and GEM, successful loading via physical adsorption into UiO-66 and UiO-66-NH_2_ pores was achieved, with N_2_ sorption measurements at 77 K indicating a decreased surface area after drug incorporation, compared against their nonloaded counterparts (Figure S4a,b). In contrast, DOX could not enter the micropores of UiO-66 due to the size disparity.^31^ Instead, it was predominantly adsorbed on the external surface and defect sites of the framework, likely due to the strong coordination between the zirconium cluster of the MOF and carbonyl groups of the anthraquinone ring of DOX, which manifests as an observable color change of the MOF from off-white to pink.^31,32^ MOF-808, which features larger pores (∼1.8 nm), facilitated more effective DOX encapsulation, resulting in a more intense MOF color change to purple upon drug loading (Figure 2c). This was supported by nitrogen sorption isotherm measurements, showing a significant decrease in the available pore space after drug loading (Figure S4c).

On the other hand, PTX, a large and highly hydrophobic molecule, could not be successfully loaded into UiO-66 and MOF-808 via postsynthetic approaches, as previous reports have shown that effective encapsulation of PTX requires MOFs with sufficiently large pores.^33,34^ Since ZIF-8 has large internal cavities but narrow aperture sizes, *in situ* encapsulation of PTX was attempted during the synthesis of ZIF-8 in methanol. Nevertheless, the resulting PTX@ZIF-8 exhibited low drug loading (∼0.3 wt %), which could be attributed to weak interactions between PTX and the ZIF-8 framework.^35^ With PTX unsuitable for delivery from the MOF carriers chosen, it was, therefore, released from the gel phase in subsequent formulations (*vide infra*).

To establish a benchmark for differential drug release using MOF–thermogel nanocomposites, we first studied the drug release performance from the MOFs in 25 mM HEPES buffer (pH 7) at 37 °C with the following combinations: GEM@UiO-66, GEM@UiO-66-NH_2_, 5-FU@UiO-66, 5-FU@UiO-66-NH_2_, and DOX@MOF-808. The HEPES buffer was chosen to simulate physiological conditions and was previously shown to maintain the structural integrity of MOFs throughout the release period.^36^ Each drug-loaded MOF sample was homogeneously suspended in 1 mL of buffer in an Eppendorf tube and incubated with gentle shaking. Cumulative drug release at defined time intervals was quantified via HPLC (see Section S4). As shown in Figure 2d, all combinations containing GEM and 5-FU exhibited an initial burst release within the first 2 h, while most of the loaded drugs within the MOFs were released within 8 h. Notably, GEM@UiO-66-NH_2_ showed a relatively slower release profile, with approximately 45% of GEM released in the first 2 h, compared to over 70% from GEM@UiO-66. The reduced release rate of GEM from UiO-66-NH_2_ is likely due to the hydrogen bonding interactions between the amino groups on the MOF and GEM molecules. A similar trend was observed for 5-FU-loaded MOFs, where the release from UiO-66-NH_2_ was slower than that from UiO-66, suggesting that linker modification can influence the release kinetics through the host–guest interaction. However, the difference was less pronounced, probably due to the smaller size of 5-FU compared to GEM, which induces faster diffusion and decreases the impact of host–guest interactions. In contrast, the release of DOX from MOF-808 in HEPES buffer (pH 7) was significantly slower, with no detectable DOX observed by HPLC over a 5-day period (Figure S14). This indicates strong interactions between DOX and the MOF-808 framework. Even under acidic conditions using acetate buffer (pH 5)—which typically enhances the DOX solubility^37^—the release remained minimal, with less than 20% of DOX detected over 5 days.

### Design of MOF-Gel Nanocomposites for Targeted Dual-Drug Combination Therapy

Having established the suitability of different MOFs for sustained release of the chemotherapeutic drugs, we then assessed the effects of adding MOFs to EPC thermogels on the macroscopic gel properties. The EPC polyurethane random multiblock copolymer was synthesized via polyaddition of hexamethylene diisocyanate (HMDI), PEG, PPG, and PCL-diol, catalyzed by dibutyltin dilaurate (DBTL).^46^ The resulting polyurethane has a number-average molecular weight (*M*_n_) of 77 kDa and a polydispersity index (PDI) of 1.9, as determined by gel permeation chromatography (GPC). As shown in the ^13^C NMR spectrum (Figure S7), only signals from urethane linkages (−NH *C* (O)­O–: 156.5 ppm) were observed, with no detectable signals corresponding to allophanates and isocyanurates, suggesting that the EPC polymer has a predominantly linear structure. At 15 wt/wt %, the gelation temperature (*T*_gel_) was determined via oscillatory temperature sweep rheological measurements to be 13.4 °C (Figure S12).

Addition of UiO-66 or UiO-66-NH_2_ (0.9 wt %) to EPC (15 wt/wt % in deionized water) retained the clear sol-to-gel transition characteristic of thermogels, with oscillatory temperature sweep rheological measurements showing *T*_gel_ below the physiological temperature in all cases (Table S2, Figure S13). This indicated that the MOF–thermogel composites could still function as injectable gel depots for drug administration at potential tumor sites. However, MOF inclusion led to a noticeable decrease in the storage modulus (*G*’) at 37 °C compared to the pristine EPC gel, suggesting a reduction in gel stiffness (Table S2). From rubber elasticity theory, this indicated that MOF addition resulted in an increase in the mesh size, which could potentially enhance the permeability and accelerate the release of the loaded drugs.^47^ To further assess the compatibility between MOFs and the EPC gel matrix, we monitored changes in the molecular weight of EPC in the presence of MOFs. The *M*_n_ of the EPC polymers remained stable over 18 days, indicating minimal polymer degradation or chain scission resulting from the MOF’s presence (Figure S3). These results demonstrate a good compatibility between MOFs and the EPC gel.

### Differential Release of Gemcitabine and Doxorubicin

Gemcitabine and doxorubicin are FDA-approved chemotherapeutics, possessing distinct mechanisms of action, and have thus been used in combination for synergistic treatment of advanced ovarian cancer and breast cancer.^48,49^ GEM is a hydrophilic nucleoside analogue that inhibits DNA synthesis and induces cell cycle arrest in the synthesis phase,^49,50^ while DOX is a relatively hydrophobic anthracycline that intercalates into DNA and inhibits topoisomerase II, leading to double-strand DNA breaks. Administering GEM prior to DOX was evaluated using the triple-negative breast cancer cell line MDA-MB-231, showing enhanced caspase activity and more effective inhibition of cell growth *in vitro*.^9,51^ Thus, to design a MOF–thermogel dual-release system that achieves faster release of GEM compared to DOX, we first evaluated the release of drugs individually in appropriate MOF–thermogel combinations.

First, we studied how the presence of the thermogel affected the drug release kinetics of GEM from GEM-loaded UiO-66 (GEM@UiO-66). UiO-66 was chosen as the carrier for GEM rather than UiO-66-NH_2_ due to the more rapid GEM release from the former, as established earlier (Figure 2d). Thus, the GEM release profiles of three formulations—GEM from EPC alone without MOF (GEM@EPC), GEM from MOF alone without gel (GEM@UiO-66), and GEM from preloaded MOFs suspended in the EPC thermogel (GEM@UiO-66/EPC)—were evaluated and are shown in Figure 3a. For preparing the drug@MOF/Gel composite, a suspension of drug-loaded MOFs in HEPES buffer was added to 15 wt/wt % EPC hydrogels in an Eppendorf tube while the gel was in its cold, sol state. Then, the mixture was equilibrated at 4 °C with intermittent vortexing to ensure homogeneous distribution of MOF particles within the gel matrix. Afterward, the MOF/Gel composite was incubated at 37 °C to achieve a sol-to-gel phase transition. The release experiment was conducted by incubating the samples in a 37 °C oven with gentle shaking and the cumulative release of GEM at different time points was determined by HPLC. The injectability of the MOF/EPC composite exhibited no alteration upon the incorporation of UiO-66 particles (Figure 3b).

**Figure 3:**
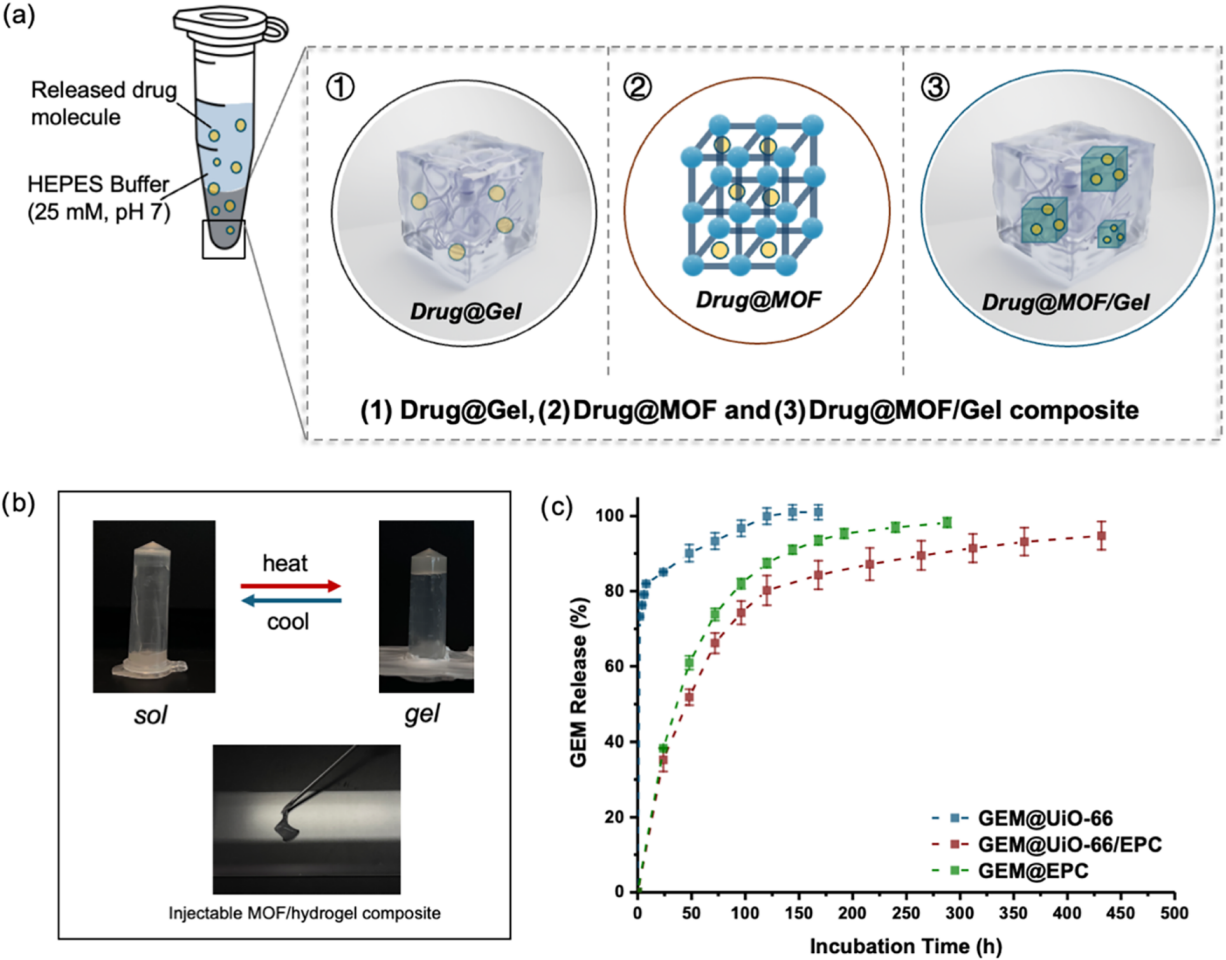
(a) Schematic illustration of the experiment setup and drug release formulations. (1) Drug-loaded EPC hydrogel (Drug@Gel), (2) drug-loaded MOF particles (Drug@MOF), and (3) hybrid composites combining drug-loaded MOFs embedded within the EPC hydrogel (Drug@MOF/Gel). (b) Photographs showing the sol–gel transition of the GEM@UiO-66/EPC composite and its injectability through a 19 G needle, illustrating the thermoresponsive and injectable properties. (c) Comparative GEM release from three combinations: GEM@UiO-66, GEM@EPC, and GEM@UiO-66/EPC (*n* = 3, error bars represent standard deviation).

As shown in Figure 3c, the GEM@UiO-66/EPC composite exhibited the most sustained release behavior of the three combinations, showing an initial rapid release within 100 h, followed by a gradual release over 18 days. This contrasted significantly with GEM@UiO-66, where approximately 70% of the drug was released within the first 2 h, followed by nearly complete release within 120 h. In comparison, complete GEM release was achieved within 250 h from GEM@EPC alone, notably without the burst release observed from GEM@UiO-66. These findings suggest that incorporating drug-loaded MOF particles into the EPC hydrogel matrix can effectively suppress burst release and significantly prolong drug release duration, which may potentially minimize systemic side effects and enhance therapeutic efficacy in anticancer treatment.

To understand how different carrier systems influence the release behavior of chemotherapeutic drugs, we applied the Korsmeyer–Peppas model, a mathematical model commonly used to describe drug release kinetics from polymeric system,^52^ to evaluate the release kinetics from the aforementioned drug formulations. The fitting was performed by using drug release data corresponding to at least 70% cumulative release. The release velocity constant (*K*) and release exponent (*n*) are summarized in Table 1. For all data sets, the correlation coefficient (*R* ^2^) exceeded 0.96 in all cases, indicating a good fit between the experimental data and the fitting equation

**Table 1:**
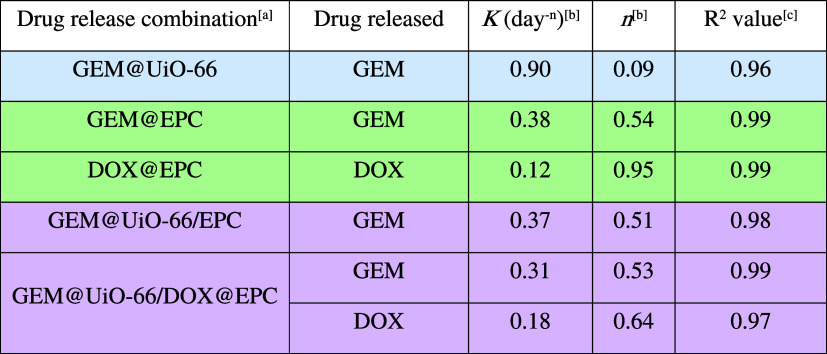
Summary of Drug Release Kinetic Parameters of GEM and DOX from Different Formulations Fitted with the Korsmeyer–Peppas Model

a Release experiment was conducted with triplicate in HEPES buffer (pH 7) at 37 °C. GEM@UiO-66 refers to GEM release from UiO-66. GEM@EPC and DOX@EPC represent GEM and DOX release from the EPC thermogel alone (without MOF), respectively. GEM@UiO-66/EPC was prepared by dispersing GEM-loaded UiO-66 particles (0.9 wt %) in chilled EPC solution (15 wt %). GEM@UiO-66/DOX@EPC was prepared by dispersing GEM-loaded UiO-66 particles (0.9 wt %) into DOX-loaded EPC solution (15 wt %) (see Section S5).

b The release velocity constant (*K*) and release exponent (*n*) were determined by fitting the Korsmeyer–Peppas model to the release profiles using data points from at least 70% cumulative release.

c *R* ^2^ values were obtained from linear regression fitting of the Korsmeyer–Peppas model.

$$\log\left(\frac{\mathrm{Mt}}{M\infty }\right)=\log K+n\log t$$

where *M_t_* is the cumulative drug amount release at time *t*, *M*∞ is the total drug amount, and *t* is the release time in days.

When using MOFs as the sole drug carrier, the drug release profile from GEM@UiO-66 in HEPES buffer (pH 7) followed a Fickian diffusion mechanism, as evidenced by the low release exponent value of 0.09. This indicates that the primary mechanism of drug release is the passive diffusion from the porous MOF framework. Changing the drug carrier from MOFs to the EPC hydrogel altered both the release rate and mechanism. For GEM@EPC, the release exponent value of 0.54 suggested a shift from purely diffusion-controlled to anomalous transport. The lower *K* value compared to that from GEM@UiO-66 indicated a slower release rate, which can be attributed to the denser gel network impeding drug diffusion. In the hybrid GEM@UiO-66/EPC formulation, a synergistic effect was observed. The presence of the gel matrix introduced additional resistance to mass transport and led to anomalous diffusion behavior (*n* = 0.51), implying both diffusion and erosion attributed to the release process. Thus, the thermogel component dominated drug release kinetics that is consistent with the initial release of GEM from UiO-66 into the EPC component, whereupon the drug was gradually released into the surrounding media as dictated by the physicochemical properties of the gel.

Interestingly, when comparing EPC gels of different concentrations (8 vs 15 wt %), we observed that a higher gel concentration unexpectedly accelerated GEM release from the GEM@UiO-66/EPC composite (Figure S15a). This is counterintuitive as denser hydrogels typically restrict diffusion due to reduced mesh sizes and increased viscosity^47^—as was indeed the case for GEM@EPC (Figure S15b). We hypothesized that the EPC gel might interact with UiO-66, possibly being taken up in the MOF internal pores and influencing drug release kinetics. To test this hypothesis, MOF particles were recovered from the GEM@UiO-66/EPC composite after the 18 days release experiment, thoroughly washed with methanol to remove any EPC polymer from the MOF surface and digested in K_3_PO_4_/D_2_O for ^1^H NMR analysis. As shown in Figure S15c, the characteristic signals corresponding to the PEG and PPG segments of the EPC polymer at 3.6 and 1.2 ppm, respectively, were observed in the digested MOF sample. This confirmed that the EPC polymer was indeed taken up by UiO-66 over time, which could have displaced the GEM molecules loaded within the MOF. The interaction between EPC and UiO-66 could have also resulted in a less stiff gel network (Table S2). The storage modulus (*G*’) at 37 °C decreased notably from 4.0 to 1.7 kPa upon inclusion of UiO-66, which may be due to the partitioning of EPC chains into MOF. These results point to a bidirectional interaction wherein the MOF can influence the gel microstructure, while the gel components in turn can alter the interaction between MOF and the encapsulated drug.

Unlike the diffusion-dominated release of GEM from the EPC gel, DOX exhibits a near-zero-order release profile (Figure S16), with release exponent *n* = 0.95 (Table 1), suggesting that the release is governed primarily by gel matrix erosion rather than passive diffusion. NMR analysis of the DOX-loaded gel showed that the aromatic proton signals of DOX exhibited a downfield shift compared with DOX alone, accompanied by a slight upfield shift of the signals corresponding to the HMDI linker of EPC (Figure S8). These observations suggest partitioning of DOX into the EPC micelles that constitute the gel matrix, likely mediated by hydrophobic and hydrogen bonding interactions.

Based on the release velocity constants obtained for GEM@UiO-66/EPC and DOX@EPC (Table 1), the clinically desired drug administration sequence of initial GEM delivery followed by DOX^9,53^ could be realized with a differential release system in which GEM is loaded into the UiO-66 component with EPC serving as the depot for DOX. Hence, the simultaneous differential release behavior of both drugs from GEM@UiO-66/DOX@EPC was investigated. As shown in Figure 4a, UiO-66-encapsulated GEM exhibits a pronounced first-order release within the first 72 h, reaching nearly 60% cumulative release and plateauing thereafter. The fast diffusion is consistent with GEM’s hydrophilic nature and the relatively weak host–guest interaction with the MOF. DOX, embedded directly within the EPC hydrogel matrix, exhibited a markedly slower, and more sustained release behavior, with only about 35% of the drug released over the same time frame as GEM and continuing gradually over several days. Notably, after almost-maximal release of GEM after 120 h, DOX release could be sustained over the next 240 h. This prolonged release of DOX is primarily attributed to its hydrophobicity and interactions with the EPC gel network (*vide supra*).

**Figure 4:**
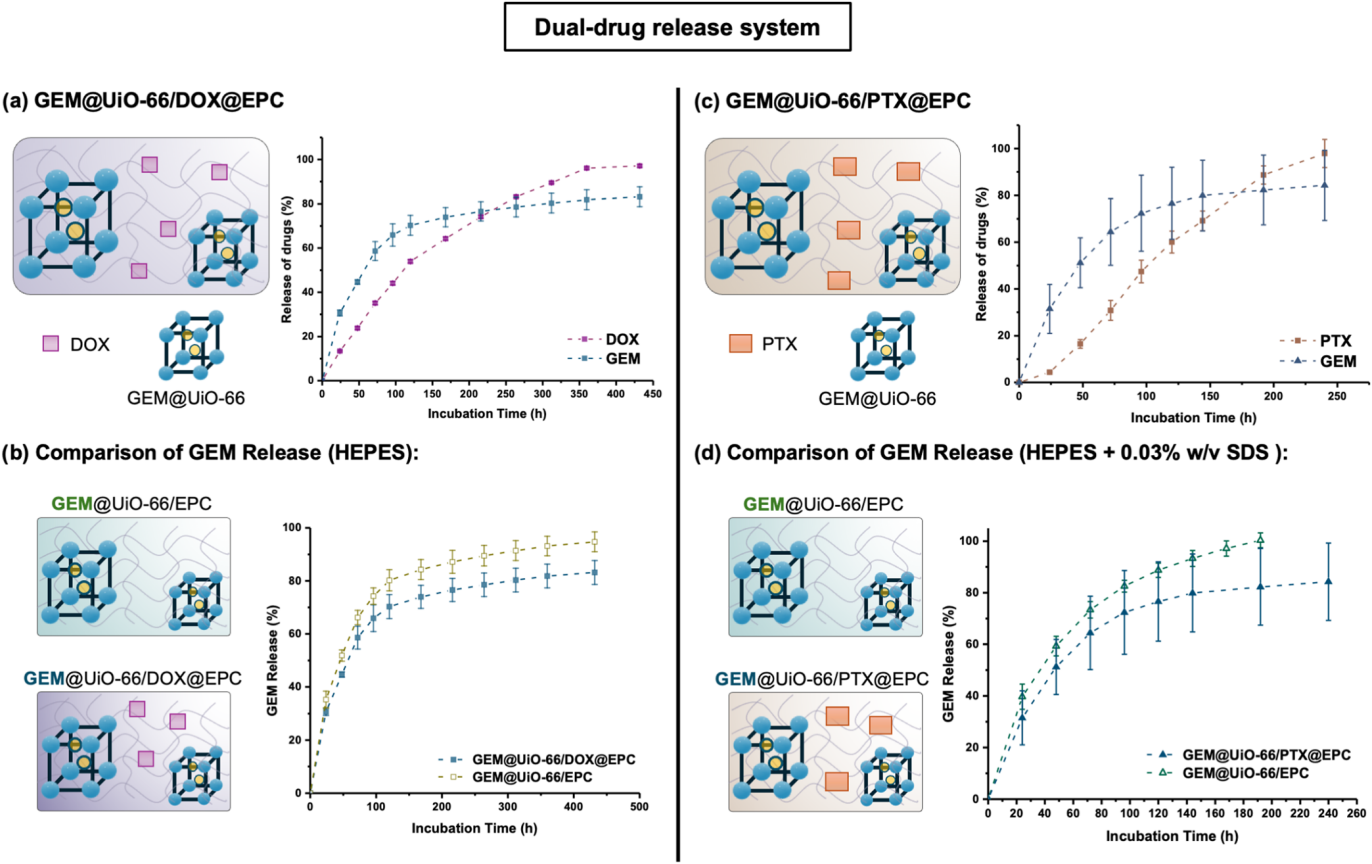
Schematic representation and cumulative drug release profile of the dual-drug release system. (a) Dual-drug release from GEM@UiO-66/DOX@EPC in HEPES (25 mM, pH 7). (b) Comparative GEM release from GEM@UiO-66/EPC and GEM@UiO-66/DOX@EPC. (c) Dual-drug release from GEM@UiO-66/PTX@EPC in HEPES (0.03 w/v% SDS). (d) Comparative GEM release from GEM@UiO-66/EPC and GEM@UiO-66/PTX@EPC.

Interestingly, the presence of DOX was also found to influence the release of GEM. A comparison of GEM release profiles between the GEM@UiO-66/EPC system and GEM@UiO-66/DOX@EPC showed a slower GEM release in the dual-drug formulation (Figure 4b). During the drug release process, a visible color change of the UiO-66 particles from white to pink was observed, indicating DOX adsorption on the external surface of the MOF. This observation was further supported by HPLC analysis of surface-bound DOX in the digested UiO-66 sample (Section S4). NaHCO_3_ digestion of UiO-66 recovered from the GEM@UiO-66/DOX@EPC composite after 18 days release period revealed approximately 3 μg of DOX, corresponding to about 6 wt % of the initial DOX content in the EPC gel. Thermogravimetric analysis (TGA) of as-synthesized UiO-66 revealed approximately 0.6% defect sites, which may facilitate DOX adsorption via defect-assisted coordination (Figure S5). These results suggested that the surface-adsorbed DOX may hinder the release of GEM from UiO-66. On the other hand, while the release kinetics of DOX from DOX@EPC and GEM@UiO-66/DOX@EPC remained largely similar over the first 120 h (Figure S16), a change in the mechanism of release from erosion-dominated in DOX@EPC (*n* = 0.95, Table 1) to anomalous transport in GEM@UiO-66/DOX@EPC (*n* = 0.64) resulted. This may have arisen from the MOF-induced weakening of the gel matrix (Table S2), which facilitated diffusion of DOX from the thermogel component. Indeed, reducing the concentration of EPC from 15 to 8 wt % in DOX@EPC led to a similar change in the release mechanism (Figure S17). These results suggest a mutual interaction among GEM@UiO-66, DOX, and the EPC matrix, collectively contributing to the differential release of GEM and DOX.

In addition, the dual-release profile observed in GEM@UiO-66/DOX@EPC closely mimics clinically beneficial pharmacokinetics. These findings underscore the capability of the MOF/gel composite to achieve a staggered, differential drug release system for synergistic combination therapy. Importantly, from the 3rd to 18th day, the daily incremental release of GEM from the composite remains below its clinically reported saturated plasma concentration of 20 μmol/L (5.26 ppm). Since GEM is a prodrug that requires intracellular phosphorylation by deoxycytidine kinase to form its active triphosphate metabolite, maintaining GEM levels below this threshold is crucial. When the GEM concentration exceeds 20 μmol/L, deoxycytidine kinase, a rate-limiting enzyme, becomes saturated, thereby limiting further activation of GEM.^54,55^ The MCF-7 breast cancer cell line, widely used *in vitro* model, has a reported half-maximal inhibition value (IC_50_) for GEM of 80 nmol/L (0.02 ppm)^3^. In our study, within the first 70% cumulative release, the daily incremental release of GEM from GEM@UiO-66/DOX@EPC ranges from 1 to 15 ppm, staying within a therapeutically effective window and exceeding the IC_50_ concentration while ensuring efficient drug activation. The IC_50_ for DOX in MCF-7 3D cell culture is approximately 10 μM (5 ppm),^56^ The observed daily DOX release from the composite ranges from 6 to 12 ppm, indicating sufficient drug availability for cytotoxic activity,

Overall, these results demonstrate that the designed MOF/gel composite GEM@UiO-66/DOX@EPC effectively delivers both drugs at therapeutically relevant levels and mimics the preferential pharmacokinetic sequences, supporting its potential for combination cancer therapy.

### Differential Release of Gemcitabine and Paclitaxel

In the treatment of advanced nonsmall-cell lung cancer (NSCLC), there is significant interest in using nonplatinum-based agents to improve therapeutic efficacy while minimizing toxicity. Both GEM and paclitaxel (PTX) are active as single agents with tolerable toxicity profiles and are commonly used as standard drugs in NSCLC treatment. Clinical reports exploring doublet regimes combining GEM and PTX demonstrated improved response rate, survival rate, disease control rate in phase II studies, and comparable efficacy in phase III randomized trials compared to platinum agent-based chemotherapy.^57^

Importantly, the scheduling of drug administration plays a critical role in maximizing pharmaceutical efficacy. In lung cancer cell lines studies including the adenocarcinoma cell line A549 and the squamous carcinoma cell line H520, the sequential delivery of GEM followed by PTX has demonstrated a synergistic effect in suppressing tumor growth.^11^ An initial dose of GEM induces cell cycle arrest in phase S of the cell cycle, thereby priming the cancer cells for subsequent exposure to PTX.^58^ Subsequent PTX exposure stabilizes microtubules during mitosis, leading to apoptosis. Motivated by this clinical relevance, we developed a MOF gel composite (GEM@UiO-66/PTX@EPC), where GEM is encapsulated in UiO-66 and PTX is loaded into the EPC hydrogel matrix, with sodium dodecyl sulfate (SDS) added as a surfactant to the HEPES buffer to solubilize the PTX within the gel component. *In vitro* drug release experiments (Figure 4c) show a distinct kinetic profile, with GEM exhibiting an initial release from the MOF, achieving 64% release in 72 h. Its fast diffusion is consistent with the anomalous transport mechanism, given that the *n* value is 0.53 (Table 2). In contrast, PTX, a bulky and highly hydrophobic compound showed a considerably slower release with a sustained pattern extending over 10 days, with only 20% released after 48 h. The release profile of PTX was best fitted to a Super Case II transport mechanism (zero-order release), suggesting the release is governed by the surface erosion of the micellar supramolecular thermogel. This indicates that PTX is tightly encapsulated within micellar cores of the EPC copolymer and can only be released following surface erosion of the thermogel.

**Table 2:**
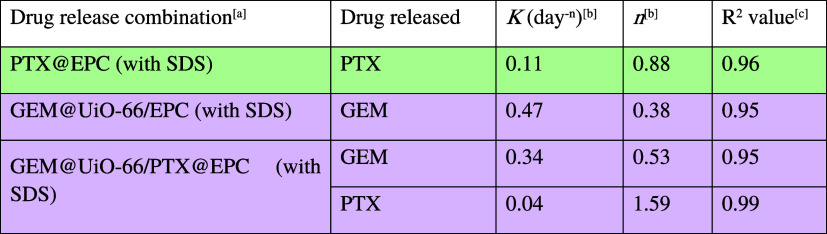
Summary of Drug Release Kinetic Parameters of GEM and PTX from Different Formulations Fitted with the Korsmeyer–Peppas Model

a Release experiment was conducted with triplicate in HEPES buffer (pH 7) with 0.03 wt/v% SDS at 37 °C. PTX@EPC represents PTX release from the EPC thermogel alone (without MOF). GEM@UiO-66/EPC was prepared by dispersing GEM-loaded UiO-66 particles (0.9 wt %) in chilled EPC solution (15 wt %). GEM@UiO-66/PTX@EPC was prepared by dispersing GEM-loaded UiO-66 particles (0.9 wt %) with PTX-loaded EPC solution (15 wt %) (see Section S5).

b The release velocity constant (*K*) and release exponent (*n*) were determined by fitting the Korsmeyer–Peppas model to the release profiles, using data points from at least 70% cumulative release.

c *R* ^2^ values were obtained from linear regression fitting of the Korsmeyer–Peppas model.

A comparison of GEM release profiles in HEPES buffer (with 0.03 w/v% SDS) between the GEM@UiO-66/EPC system and the EPC gel loaded with PTX is shown in Figure 4d. The presence of PTX in the formulation led to a notably slower GEM release (Table 2). As shown in Table S1, the presence of PTX in EPC (in 0.03 wt/v% SDS) was found to slightly increase the storage modulus (*G*’) compared to EPC alone in the same buffer system. This suggests that interactions between PTX and the gel matrix may enhance the structural integrity of the gel, thereby slowing GEM diffusion.

Like the dual-release combination of GEM+DOX, the release kinetics of GEM and PTX from the GEM@UiO-66/PTX@EPC composite mimics the clinically relevant sequential release profile of GEM, followed by PTX. The initial, more rapid release of GEM is accompanied by very low quantities of PTX released (<48 h); however, PTX release is sustained even when GEM release has ceased (>96 h) (Figure 4c). Compared with the recorded IC_50_ values of GEM for A549 cell line as 6.6 nM (1 × 10^–3^ ppm) and that of PTX as 46.1 nM (0.04 ppm),^11^ the daily incremental release of GEM and PTX in the dual-drug-loaded MOF–hydrogel composite within the first 70% cumulative release ranged from 1 to 16 ppm and 6–10 ppm, respectively, indicating the potential to effectively inhibit cancer cell growth.

### MOF–Thermogel Nanocomposites for Triple-Drug Combination Therapy

After establishing that MOF–thermogel composites can provide a suitable platform for achieving dual-drug differential release, we further investigated the possibility of designing a composite for achieving differential release of three simultaneously loaded drugs. To this end, we selected a triple-drug combination comprising GEM, DOX, and 5-fluorouracil (5-FU), driven by their representative clinical relevance and synergistic interactions in various cancer therapies.^59^

As a pyrimidine analogue, 5-FU can inhibit thymidylate synthase, disrupt nucleoside metabolism, and be misincorporated into RNA and DNA, ultimately triggering cytotoxicity and cell apoptosis.^60^ Clinical studies have demonstrated that combining 5-FU with GEM yields synergistic antitumor activity in several cancer types, including pancreatic, colorectal, and biliary tract carcinomas.^61,62^ Moreover, the synergy of 5-FU+GEM is sequence-dependent, whereby pretreatment with 5-FU has been reported to enhance both the cellular uptake and therapeutic efficacy of GEM.^63,64^ This strategy also helps mitigate the dose-limiting toxicity of GEM to normal cells, which arises from its rapid plasma deamination and consequent short half-life (8–17 min).^65^

The coadministration of 5-FU and DOX has also been shown to enhance antiproliferative effect and apoptosis at lower doses in breast cancer models, particularly in triple-negative breast cancer (TNBC), where monotherapies are often insufficient.^66,67^ In recent *in vivo* studies, the combination treatment of DOX+5-FU induced broad metabolic disruptions across several key pathways including purine, pyrimidine, β-alanine, and sphingolipid metabolism.^66^

Given that both GEM and 5-FU are hydrophilic drugs, as indicated by their partition coefficients in Figure 2a, we anticipated minimal difference in their release profiles when the drugs were loaded into the EPC gel. Indeed, simultaneous loading of 5-FU and GEM into the EPC gel (15 wt %) resulted in a similar release, indicating no temporal control between the two drugs (Figure S18). Thus, to design a suitable triple-release system with descending release order of 5-FU > GEM > DOX, we built upon our earlier release studies showing a clear temporal separation between GEM and DOX release. Based on this, we strategically loaded 5-FU and DOX into the EPC gel while encapsulating GEM with UiO-66 to slow its release relative to 5-FU.

As shown in Figure 5a, the release profiles of 5-FU, GEM, and DOX from the GEM@UiO-66/5-FU+DOX@EPC triple-drug-loaded composite in HEPES buffer showed that 5-FU was most rapidly released, followed by a slower release of GEM, with DOX being released the slowest. The release of GEM remains governed by anomalous transport (*n* = 0.49, Table 3), like that from GEM@UiO-66/DOX@EPC (Table 1), with similar *K* values, implying that the presence of the third drug 5-FU within the EPC gel matrix does not significantly interfere with the GEM release kinetics (Figure 5b). Also, the release profile of 5-FU closely resembles that observed in EPC alone, with both showing diffusion-driven release behavior (Table 3, Figure 5c). The weak interaction between 5-FU and MOF might be due to the lack of strongly coordinating sites on the 5-FU molecule. In contrast, the DOX release, though significantly slower than that from the MOF-free 5-FU+DOX@EPC (Figure 5d), shows Korsmeyer–Peppas release parameters similar to that from the dual-release GEM@UiO-66/DOX@EPC composite (Table 1). This suggests that similarly, DOX is also coordinating on the external surface of UiO-66. From a pharmacological perspective, the IC_50_ of 5-FU against MCF-7 breast cancer cells has been reported as 140.2 μM (18 ppm).^68^ In this study, within the first 70% cumulative release, the daily incremental concentration from the triple-drug formulation matched the therapeutic windows as GEM at 1–10 ppm, DOX at 5–14 ppm, and 5-FU at 10–30 ppm. Successfully achieving differentiated release of multiple drugs simultaneously through the easily formulated MOF–thermogel composite platform highlights its versatility for tailoring multidrug release and underscores its potential for further clinical translation, where staggered release timing and codelivery can enhance therapeutic efficacy, reduce systemic toxicity, and minimize drug resistance.

**Figure 5:**
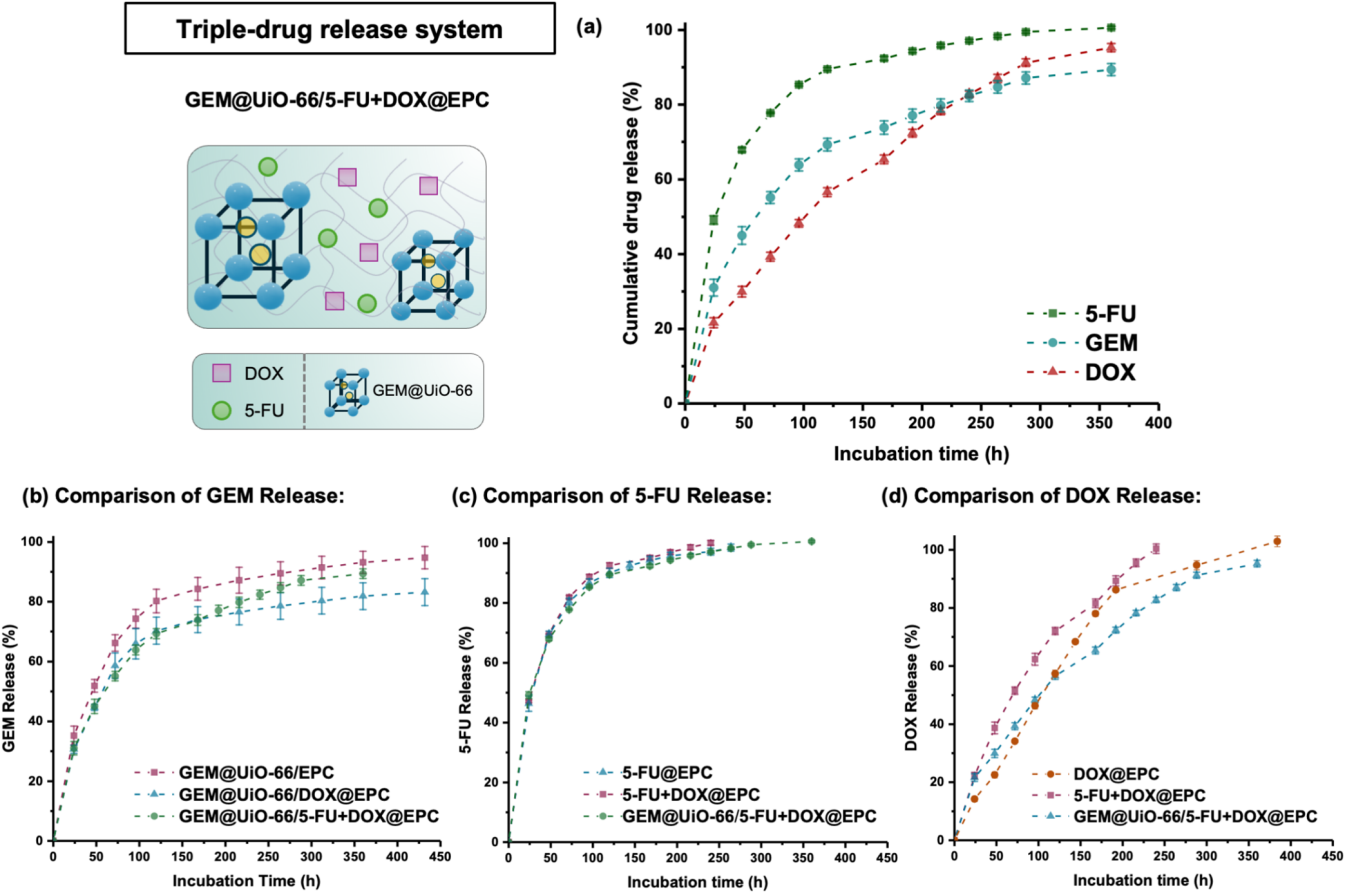
(a) Schematic representation and cumulative drug release profile of the multidrug release system GEM@UiO-66/5-FU+DOX@EPC. (b) Comparison of GEM release in three formulations. (c) Comparison of 5-FU release in three formulations. (d) Comparison of DOX release in three formulations.

**Table 3:**
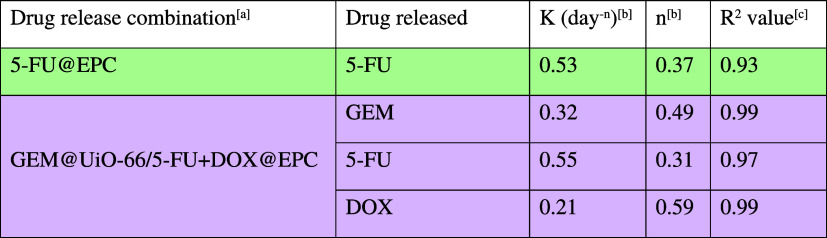
Summary of Drug Release Kinetic Parameters for GEM@UiO-66/5-FU+DOX@EPC Fitted with the Korsmeyer–Peppas Model

a Release experiment was conducted with triplicate in HEPES buffer (pH 7) at 37 °C. 5-FU@EPC represents 5-FU release from the EPC thermogel (15 wt %) alone (without MOF). GEM@UiO-66/5-FU+DOX@EPC was prepared by dispersing GEM-loaded UiO-66 particles (0.9 wt %) with 5-FU and DOX-coloaded EPC solution (15 wt %) (see Section S5).

b The release velocity constant (*K*) and release exponent (*n*) were determined by fitting the Korsmeyer–Peppas model to the release profiles, using data points from at least 70% cumulative release.

c *R* ^2^ values were obtained from linear regression fitting of the Korsmeyer–Peppas model.

## Conclusions

In conclusion, we herein demonstrated a simple yet versatile MOF–thermogel nanocomposite platform that allows sequence-designable, differentiated, and sustained release of chemotherapeutic drugs for combination multidrug solid tumor treatment. From preliminary *in vitro* drug release studies that characterized the physicochemical behavior and release kinetics of various drug-loaded formulations, e.g., drug@MOF, drug@EPC, and drug@MOF/EPC, different combinations of drugs and carriers were designed to achieve clinically relevant dosing sequences. Sequential and differentiated release of the dual-drug combinations (GEM → DOX and GEM → PTX) were achieved by encapsulating GEM in the MOF and DOX/PTX in the gel components, respectively, leveraging on distinct differences in hydrophobicity, molecular size, and encapsulation behavior, enabling sustained drug release over at least 10 days. Different components of the multidrug formulation, namely, drugs, gel, and MOF carriers, were found to engage in mutual interactions that while not affecting the overall sequence of drug release, could alter the release kinetics and mechanical stability of the system. Such interactions have important implications for the long-term predictability and robustness of the drug delivery system. Furthermore, the modular nature of the MOF–thermogel nanocomposite was expanded to a triple-drug system, GEM@UiO-66/5-FU+DOX@EPC, which successfully demonstrated a clinically relevant differential release of 5-FU → GEM → DOX. In all cases, the presence of MOFs retained the characteristic thermogelling behavior of the bulk EPC thermogel phase, while enabling injectability for site-specific drug delivery, potentially to target tumor sites.

Although these simple-to-formulate MOF–thermogel multidrug release systems hold promise for overcoming the limitations of single-agent therapies and pave the way for more effective and personalized cancer therapeutics, several challenges must still be addressed. In this study, *in vitro* release experiments were conducted in buffered media to simulate physiological pH; however, the actual tumor microenvironment is more complex. It features acidic pH gradients, reactive oxygen species (ROS), overexpression of specific enzymes, hypoxia, and increased adenosine-5′-triphosphate (ATP) levels.^69^ These factors may destabilize MOF structures, alter gel erosion dynamics, or influence drug–MOF and drug–gel interactions. Further investigation using simulated biological fluids or *in vivo* models will be essential to assess the performance and stability of the MOF–thermogel nanocomposite under physiologically relevant conditions. In addition, although hydrogels can enhance the biocompatibility of MOFs,^19^ the *in vivo* cytotoxicity of MOFs remains underexplored. A comprehensive understanding of their biodistribution, bioaccumulation in tissues, metabolism, and clearance is essential before translation to clinical settings. Additionally, while the MOFs used in this study, in particular UiO-66, can be synthesized at scale,^70^ manufacturing of MOF–hydrogel/thermogel composites compatible with current good manufacturing practices (cGMPs) remains thus far unrealized. Nonetheless, our findings showcase the exciting potential of MOF–hydrogel platforms for the codelivery of multiple chemotherapeutic agents with controlled, differential release by strategic drug loading and compartmentalization.

## Supplementary Material



## Notes

XRD and nitrogen sorption measurements of MOFs, NMR spectrum of EPC gel and drug-loaded EPC gel, rheological measurement, and additional drug release (PDF)

## Abbreviations

ATP: adenosine-5′-triphosphate
5-FU: 5-fluorouracil
DK: deoxycytidine kinase
DOX: doxorubicin
FDA: food and drug administration
GEM: gemcitabine
HEPES: 4-(2-hydroxyethyl)-piperazine-1-ethanesulfonic acid
MOF: metal–organic framework
PCL: poly­(ε-caprolactone)-diol
PEG: poly­(ethylene glycol)
PPG: poly­(propylene glycol)
PTX: paclitaxel
ROS: reactive oxygen species
