# Supplementary material for: Designing MOF–Thermogel Nanocomposites for Differential Multidrug Release in Combination Cancer Therapy
Source: ACS Appl Nano Mater. 2025 Aug 24;8(35):17297–310. doi: 10.1021/acsanm.5c03527 (PMC13073680; doi:10.1021/acsanm.5c03527)
Supplement: Supplementary file 1 [file an5c03527_si_001.pdf]

Supporting Information

Designing MOF-Thermogel Nanocomposites for Differential Multidrug Release in Combination Cancer Therapy

Wenyi Zeng,[a,b] *Tristan T. Y. Tan**,[a] *Qianyu Lin,*[a] *Wei Wei Loh,*[a] *Yan Hui Lee,*[a] *Michael R. Reithofer,*[b] *Xian Jun Loh**,[a,c] *Jia Min Chin**[d] *and Jason Y. C Lim**[a,c]

AUTHOR ADDRESS

[a] Institute of Materials Research and Engineering (IMRE), Agency for Science, Technology and Research (A*STAR), 2 Fusionopolis Way, Innovis #08-03, Singapore 138634, Republic of Singapore

[b] Institute of Inorganic Chemistry, Faculty of Chemistry, University of Vienna, Währinger Str. 42, 1090 Vienna, Austria

[c] Department of Materials Science and Engineering, National University of Singapore (NUS), 9 Engineering Drive, Singapore 117576, Republic of Singapore

[d] Institute of Functional Materials and Catalysis, Faculty of Chemistry, University of Vienna, Währinger Str. 42, 1090 Vienna, Austria

Email:

Jason Y. C. Lim: jason_lim@imre.a-star.edu.sg

Jia Min Chin: jiamin.chin@univie.ac.at

Tristan T. Y. Tan: tristan_tan@imre.a-star.edu.sg

Xian Jun Loh: lohxj@imre.a-star.edu.sg

Table of Contents

S1 Materials and method 3

S2 Characterization of MOFs.. 5

S3 NMR spectra of EPC polymer 10

S4 Determination of the drug loading via HPLC 11

S5 Preparation of drug loaded hydrogel...12

S6 NMR study of drug loaded EPC..14

S7 Rheological measurement 18

S8 Additional drug release studies 20

S9 Calibration curve of GEM, DOX, 5-FU..23

S10 Korsmeyer-Peppas fitting for the release kinetic 26

Reference 37

S1 Materials and Methods

All chemicals were purchased from commercial sources and used without further purification unless mentioned. Terephthalic acid was purchased from Alfa Aesar, 5-fluorouracil and gemcitabine hydrochloride were purchased from TCI. Paclitaxel was purchased from Yunnan Hande Bio tech Co., Ltd. $CDCl_3$ and D_2O were purchased from Cambridge Isotope Laboratories. Zirconium chloride, zinc acetate, 2-methylimidazole, 2-aminoterephtalic acid, aluminum nitrate nonahydrate, poly(ethylene glycol) (PEG, 2050 Da), cetyltrimethylammonium bromide (CTAB), doxorubicin hydrochloride, phosphorus pentoxide, poly(propylene glycol) (PPG, 2000 Da), poly(ε-caprolactone)-diol (PCL, 2000 Da), hexamethylene diisocyanate (HMDI) 99%, dibutyltin dilaurate 95%, trimesic acid, acetic acid, *N,N*-dimethylformamide and 4-(2-hydroxyethyl)piperazine-1-ethanesulfonic acid (HEPES), were purchased from Sigma Aldrich. Anhydrous toluene and anhydrous diethyl ether are sourced from TEDIA. Toluene was further dried and stored over pre-activated 4 Å molecular sieves.

X-ray diffraction (XRD) data were collected using a Bruker D8-Advance X-ray diffractometer with Cu Kα (λ = 1.5406 Å) radiation, at a resolution of 0.02° and a scan rate of 0.1 s/point.

Scanning electron microscopy (SEM) images of the MOF particles were captured on a JEOL JSM6700F with 5.0 kV accelerating voltage and 10 µA emission current. The sample powder was loaded on a carbon tape and sputter coated with gold before measurement.

Nuclear magnetic resonance (NMR) was recorded with a JEOL 500 MHz spectrometer (Tokyo, Japan) in $CDCl_3$ or in D_2O. The chemical shift was measured in ppm.

Thermogravimetric analysis (TGA) was carried out in TGA 55 (TA Instruments) at a heating rate of 5 °C/min, from room temperature to 900 °C. About 20 mg sample was placed in a platinum pan.

Nitrogen adsorption-desorption isotherm was recorded by using a Micromeritics ASAP 2460 Analyzer. Prior to the measurements, the samples were degassed at 0.01 mbar, 150 °C for 10 h. For drug loaded MOF sample, the samples were degassed at 0.01 mbar, 100 °C for 10 h.

Gel permeation chromatography (GPC) was performed using the Agilent 1260 Infinity II GPC/SEC System with refractive index detector and calibration was performed using monodisperse polystyrene standards. THF was using as the eluent. 3 mg of MOF samples (UiO-66, UiO-66-NH_2) was mixed with the EPC-gel (15 wt% in deionized water) under 4 °C in an Eppendorf tube, respectively. Then the mixture was placed in an oven at 37 °C forming a gel. After a certain time, 4 mg aliquot of the mixture was taken out from the sample tube and dissolved in 1 mL THF, filtered through a 0.45 µm PTFE membrane filter before GPC measurement.

High-performance liquid chromatography (HPLC) analysis: A reverse-phase HPLC method with SHIMADZU C18 column (4.6 x 250 mm length, particle size of 5 µm) was used for the quantification of drug release. The temperature of the column oven was set at 35 °C and the injection

volume was 10 μL. All standard solutions were filtered through a 0.45 μm PTFE membrane filter before analysis. Peak identification was done by matching the retention time of the chromatographic peaks as well as the UV-Vis spectrum of the test sample with the pure standards. Quantification was estimated using external calibration method.

Rheological measurements were carried out using a TA Instruments Discovery DHR-3 hybrid rheometer equipped with 20 mm flat plate geometry and a temperature-controlled Peltier base plate. Temperature sweep measurements were performed in the range of 4 - 45 °C with a heating rate of 3 °C min^{-1}, a fixed strain of 1% and a frequency of 1 Hz. The temperature at which storage modulus first exceeded the loss modulus was recorded as the gelation temperature. Oscillatory strain sweep measurement showing the variations of G', G" and elasticity loss factor Tan (delta) with strain amplitude between 0.2% and 200% at 37 °C and a constant frequency of 1 Hz.

S2 Characterization of MOFs

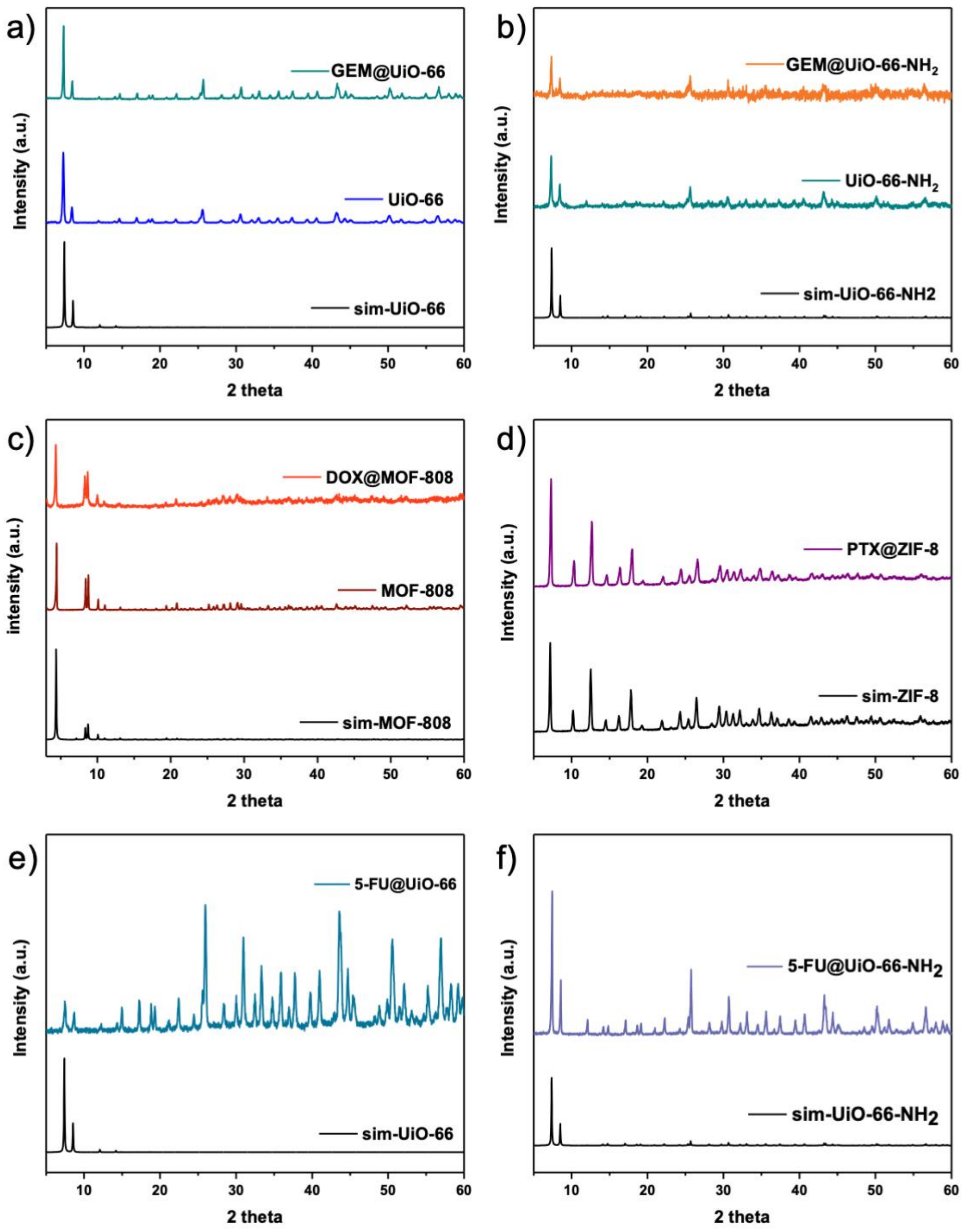

Figure S1: PXRD pattern of the as-synthesized MOF, drug-loaded MOF and simulated powder pattern obtained from reported single crystal data (in black)[1-3]. a) UiO-66 and GEM@UiO-66, b) UiO-66-NH_2 and GEM@UiO-66-NH_2, c) MOF-808 and DOX@MOF-808, d) ZIF-8 and PTX@ZIF-8, e) 5-FU@UiO-66, f) 5-FU@UiO-66-NH_2.

The structure and morphology of the particles were analyzed by field-emission scanning electron microscope (FESEM).

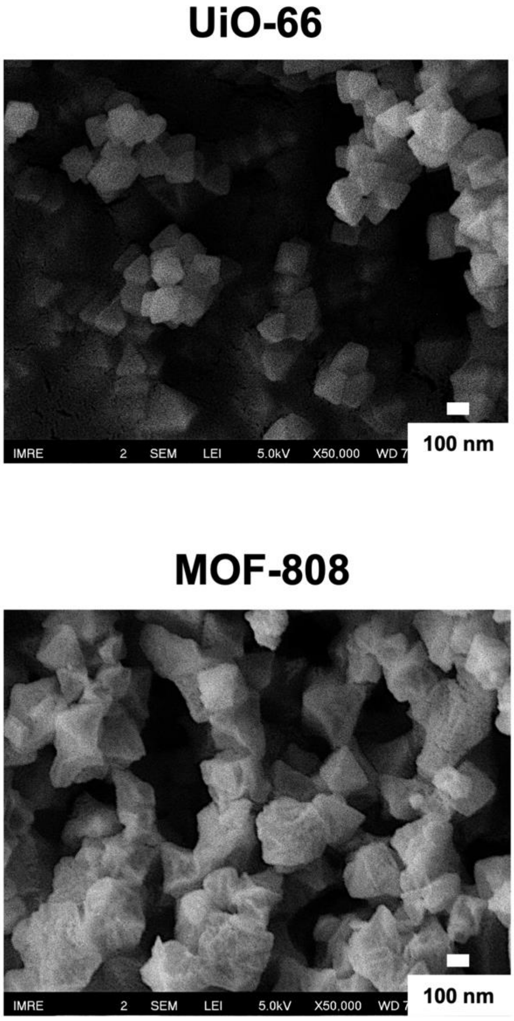

Figure S2: SEM image of the as-synthesized UiO-66, UiO-66-NH_2, MOF-808 and PTX@ZIF-8 particles.

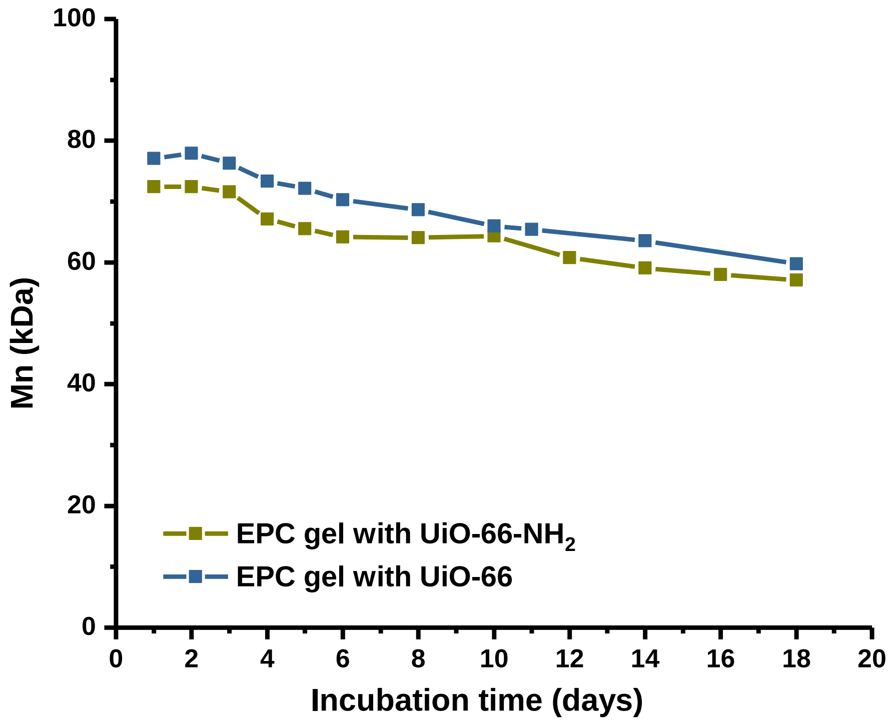

Figure S3: The number-average molecular weight (Mn) of EPC gel (15 wt/wt% in DI H_2O) in the presence of UiO-66 (3 mg) and UiO-66-NH_2 (3 mg), respectively.

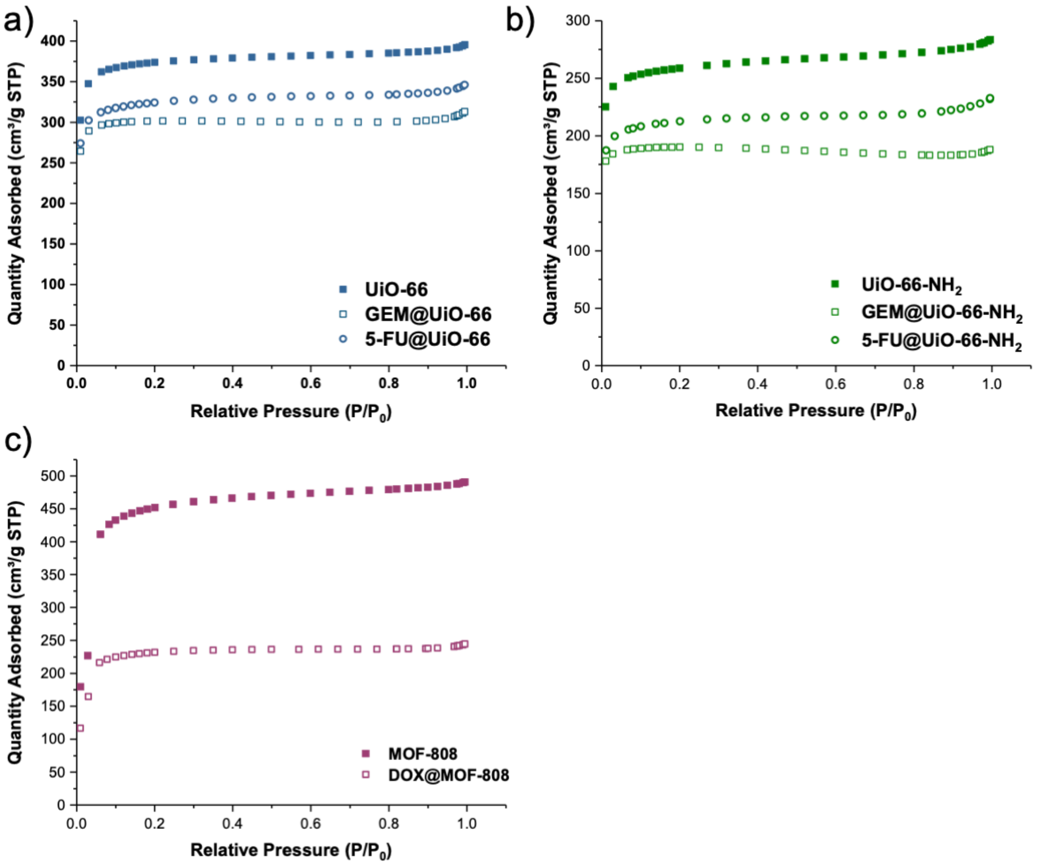

Figure S4: Nitrogen sorption isotherm measurements of MOFs and drug-loaded MOFs, showing the overall N_2 uptake decreased after drug encapsulation. a) UiO-66, 5-FU@UiO-66 and GEM@UiO-66, b) UiO-66-NH_2, 5-FU@UiO-66-NH_2 and GEM@UiO-66-NH_2, c) MOF-808 and DOX@MOF-808.

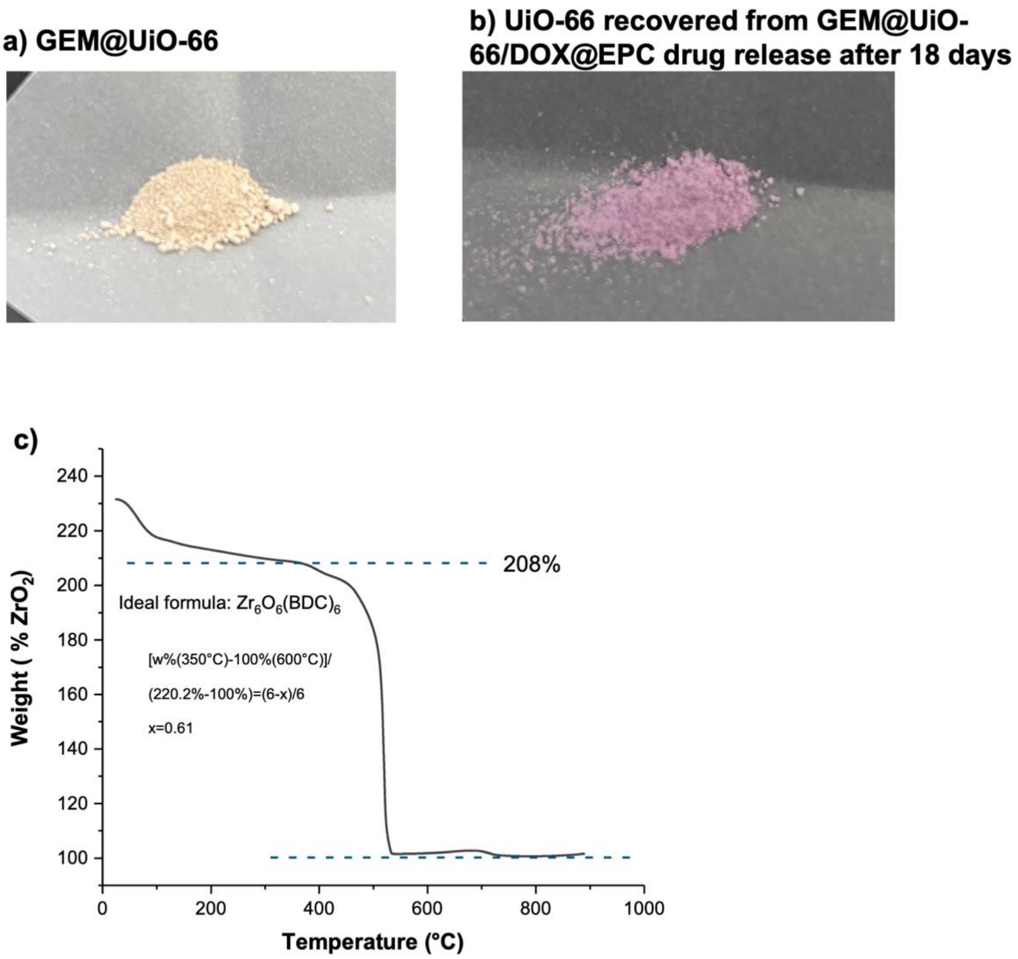

Figure S5: a) Photograph of as-synthesized GEM@UiO-66, b) Photograph of UiO-66 recovered from GEM@UiO-66/DOX@EPC, c) TGA profile of as-synthesized UiO-66 with 0.61% defect sites.

S3 NMR spectra of EPC polymer

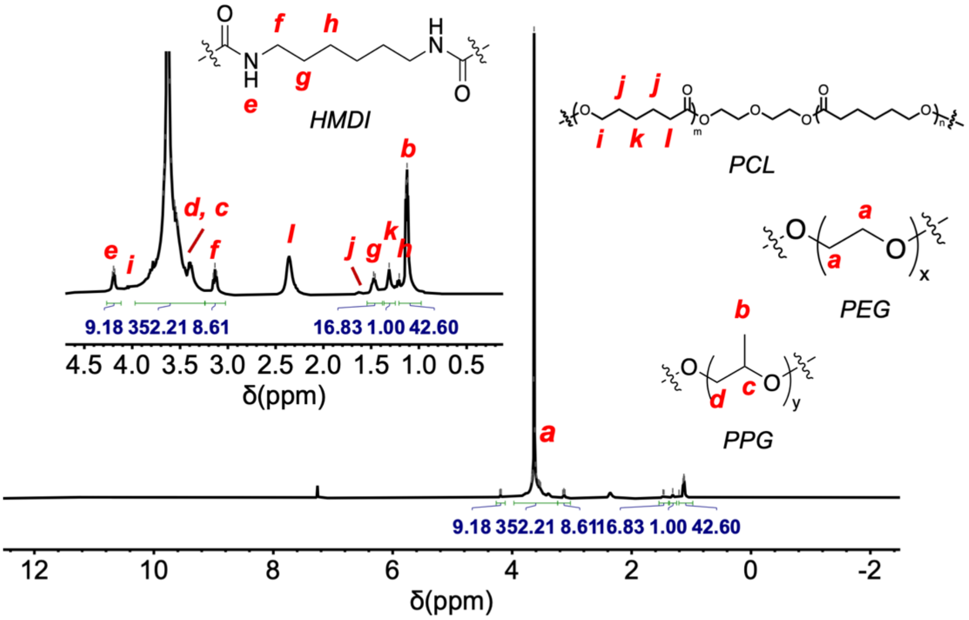

Figure S6 ^{1}H-NMR spectrum (500 MHz, $CDCl_3$) of the synthesized EPC polymer.

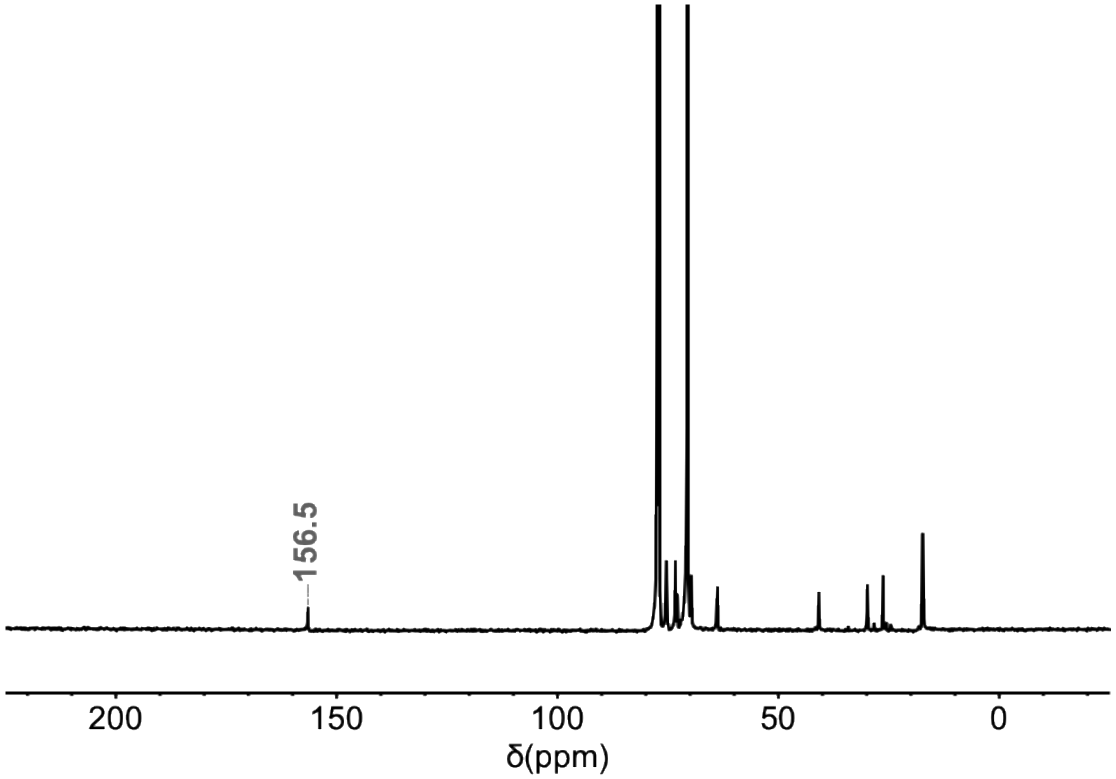

Figure S7: ^{13}C-NMR spectrum (500 MHz, $CDCl_3$) of the synthesized EPC polymer, showing the urethane carbonyl carbon at 156 ppm, with no detectable signals associated with allophanate linkages. This suggests that the EPC network is predominantly linear in structure.

S4 Determination of the drug loading via HPLC

Drug-loaded MOFs were prepared by the impregnation of as-synthesized MOF particles with drug solution under stirring at room temperature. The encapsulated drug concentration was quantified by high-performance liquid chromatography (HPLC).

For gemcitabine (GEM): 5 mg of GEM@UiO-66 and GEM@UiO-66-NH_2 were added with ammonium bicarbonate (10 mg) into 1 mL DI H_2O, separately. The mixture was sonicated until completely dissolved and forming a colorless solution. Then the solution was filtered through a 0.45 μm PTFE membrane filter and submitted for HPLC measurement.

For doxorubicin (DOX): 5 mg of DOX@MOF-808 was added with ammonium bicarbonate (25 mg) into 1 mL DI H_2O, separately. The mixture was sonicated until completely dissolved and forming a colorless solution. Then the solution was filtered through a 0.45 μm PTFE membrane filter and submitted for HPLC measurement.

For paclitaxel (PTX): PTX@ZIF-8 (5 mg) was charged in 1 mL DI H_2O (10 v/v% HCl) and sonicated until forming a colorless solution. Then the solution was filtered through a 0.45 μm PTFE membrane filter and submitted for HPLC measurement.

For 5-fluorouracil (5-FU): 5 mg of 5-FU@UiO-66 and 5-FU@UiO-66-NH_2 were added with ammonium bicarbonate (10 mg) into 1 mL DI H_2O, separately. The mixture was sonicated until completely dissolved and forming a colorless solution. Then the solution was filtered through a 0.45 μm PTFE membrane filter and submitted for HPLC measurement.

For characterizing the DOX loaded onto UiO-66 recovered from the GEM@UiO-66/DOX@EPC composite after drug release, the MOF particles were separated from the EPC gel in individual Eppendorf tubes by repeated washing with methanol, centrifugation, and drying at 60 °C at the end. After drying, the resulting pink UiO-66 particles were added with ammonium bicarbonate (10 mg) into 1 mL DI H_2O. The mixture was sonicated until completely dissolved, then filtered through a 0.45 μm PTFE membrane filter and submitted for HPLC measurement. The DOX concentration of the triplicate samples was determined to be 3.3 μg, 1.6 μg and 3.8 μg, respectively.

HPLC methods for quantification of the drug release:

1. GEM release: Flow rate is 0.8 mL/min, solvent mixture is DI H_2O:Acetonitrile (ACN) 50:50. The gemcitabine was detected at 267 nm with the retention time at 3.28 min.
2. DOX release: Flow rate is 1 mL/min, solvent mixture is DI H_2O (pH 3, phosphoric acid): ACN 45:55. The doxorubicin was detected at 480 nm with the retention time at 2.69 min.
3. PTX release: Flow rate is 1 mL/min, solvent mixture is DI H_2O: ACN 30:70. The paclitaxel was detected at 227 nm with the retention time at 5.1 min.
4. 5-FU release: Flow rate is 0.8 mL/min, solvent mixture is DI H_2O (pH 3, phosphoric acid): MeOH 90:10. The 5-FU was detected at 265 nm with the retention time at 5.5 min.
5. DOX quantification from digested MOF samples using NH_4HCO_3: Flow rate is 0.8 mL/min, solvent mixture is DI H O: ACN 70:30. The doxorubicin was detected at 480 nm with the retention time at 4.9 min.

S5 Preparation of drug loaded hydrogel

All drug-loaded EPC hydrogels were prepared in triplicates in 2.5 mL Eppendorf tube and 1 mL HEPES buffer was added above the gel layer.

GEM@EPC: 70 mg EPC gel was weighed and dissolved in 300 µL DI H_2O at 4 °C overnight. 100 µL stock solution of GEM (0.5 mg/mL in HEPES buffer) was added to EPC-Gel and equilibrated at 4 °C overnight. Afterwards, the gel mixture was warmed at 37 °C to form a gel.

5-FU@EPC: 70 mg EPC gel was weighed and dissolved in 300 µL DI H_2O at 4 °C overnight. 100 µL stock solution of 5-FU (0.5 mg/mL in HEPES buffer) was added to EPC-Gel and equilibrated at 4 °C overnight. Afterwards, the gel mixture was warmed at 37 °C to form a gel.

DOX@EPC: 70 mg EPC gel was weighed and dissolved in 300 µL DI H_2O at 4 °C overnight. 100 µL stock solution of DOX (0.5 mg/mL in HEPES buffer) was added to EPC-Gel and equilibrated at 4 °C overnight. Afterwards, the gel mixture was warmed at 37 °C to form a gel.

PTX@EPC: 70 mg EPC gel was weighed and dissolved in 390 µL DI H_2O at 4 °C overnight. 10 µL stock solution of PTX (5 mg/mL in ethanol) was added to EPC-Gel and equilibrated at 4 °C overnight. Afterwards, the gel mixture was warmed at 37 °C to form a gel.

GEM@UiO-66/EPC: 70 mg EPC gel was weighed and dissolved in 200 µL DI H_2O at 4 °C overnight. 200 µL of GEM@UiO-66 stock suspension in HEPES buffer (3 mg/mL) was added to EPC-Gel solution. The mixture was equilibrated at 4 °C overnight. Afterwards, the gel mixture was warmed at 37 °C to form a gel.

GEM@UiO-66/DOX@EPC: 70 mg EPC gel was weighed and dissolved in 100 µL DI H_2O at 4 °C overnight. 200 µL of GEM@UiO-66 stock suspension in HEPES buffer (3 mg/mL) and 100 µL stock solution of DOX (0.5 mg/mL in HEPES buffer) were added to EPC-Gel solution. The mixture was equilibrated at 4 °C overnight. Afterwards, the gel mixture was warmed at 37 °C to form a gel.

GEM@UiO-66/PTX@EPC: 70 mg EPC gel was weighed and dissolved in 190 µL DI H_2O at 4 °C overnight. 200 µL of GEM@UiO-66 stock suspension in HEPES buffer (3 mg/mL) and 10 µL stock solution of PTX (5 mg/mL in ethanol) were added to EPC-Gel solution. The mixture was equilibrated at 4 °C overnight. Afterwards, the gel mixture was warmed at 37 °C to form a gel.

GEM@UiO-66/5-FU+DOX@EPC: 70 mg EPC gel was weighed and dissolved in 100 µL DI H_2O at 4 °C overnight. 200 µL of GEM@UiO-66 stock suspension in HEPES buffer (3 mg/mL), 50 µL stock solution of DOX (1 mg/mL in HEPES buffer) and 50 µL stock solution of 5-FU (1 mg/mL in HEPES buffer) were added to EPC-Gel solution. The mixture was equilibrated at 4 °C overnight. Afterwards, the gel mixture was warmed at 37 °C to form a gel.

GEM+DOX@EPC: 70 mg EPC gel was weighed and dissolved in 300 µL DI H_2O at 4 °C overnight. 50 µL stock solution of DOX (1 mg/mL in HEPES buffer) and 50 µL stock solution of GEM (1 mg/mL in HEPES buffer) were added to EPC-Gel and equilibrated at 4 °C overnight. Afterwards, the gel mixture was warmed at 37 °C to form a gel.

5-FU+DOX@EPC: 70 mg EPC gel was weighed and dissolved in 300 µL DI H_2O at 4 °C overnight. 50 µL stock solution of DOX (1 mg/mL in HEPES buffer) and 50 µL stock solution of 5-FU (1 mg/mL in HEPES buffer) were added to EPC-Gel and equilibrated at 4 °C overnight. Afterwards, the gel mixture was warmed at 37 °C to form a gel.

GEM@UiO-66: 200 µL of GEM@UiO-66 stock suspension in HEPES buffer (3 mg/mL) was added in 800 µL HEPES buffer.

GEM@UiO-66-NH_2: 200 µL of GEM@UiO-66-NH_2 stock suspension in HEPES buffer (3 mg/mL) was added in 800 µL HEPES buffer.

5-FU@UiO-66: 200 μL of 5-FU@UiO-66 stock suspension in HEPES buffer (3 mg/mL) was added in 800 μL HEPES buffer.
5-FU@UiO-66-NH$_2$: 200 μL of 5-FU@UiO-66-NH$_2$ stock suspension in HEPES buffer (3 mg/mL) was added in 800 μL HEPES buffer.
DOX@MOF-808: 200 μL of DOX@MOF-808 stock suspension in HEPES buffer (3 mg/mL) was added in 800 μL HEPES buffer (pH 5). For acetate buffer: 200 μL of DOX@MOF-808 stock suspension in acetate buffer (3 mg/mL) was added in 800 μL acetate buffer (pH 5).

The triplicated samples were incubated at 37 °C oven under continuous shaking. After a certain time, 0.8 mL of an aliquot of supernatant was extracted from the sample tube containing EPC and MOF-EPC, followed by addition of fresh 0.8 mL 25 mM HEPES buffer. For the MOF samples, 0.8 mL from the liquid part was isolated from the MOF-buffer suspension by centrifugation. Afterwards, 0.8 mL fresh HEPES buffer was replaced. In the case of using PTX, the buffer system was replaced by 25 mM HEPES buffer with 0.03 w/v% SDS.

S6 NMR study of drug loaded EPC

NMR sample preparation in D_2O: for the pure drug controls, 5 mg of drug (GEM, DOX, 5-FU) was dissolved in 500 µL of D_2O to form a clear solution, respectively. For PTX, 5 mg of PTX and 17 mg SDS were added in 500 µL D_2O, resulting in a uniformly dispersed emulsion.

For the blank control, 5 mg of EPC gel was dissolved in 500 µL D_2O.

For the EPC+ SDS control, 5 mg of EPC gel was added with 17 mg SDS and dissolved in 500 µL D_2O.

For drug-loaded EPC sample: 5 mg of drug (GEM, DOX, 5-FU) was mixed with 5 mg of EPC and dissolved in 500 µL D_2O, respectively. In the case of PTX, 5 mg of PTX, 5 mg of EPC and 17 mg SDS were combined in 500 µL D_2O, forming a uniformly dispersed emulsion.

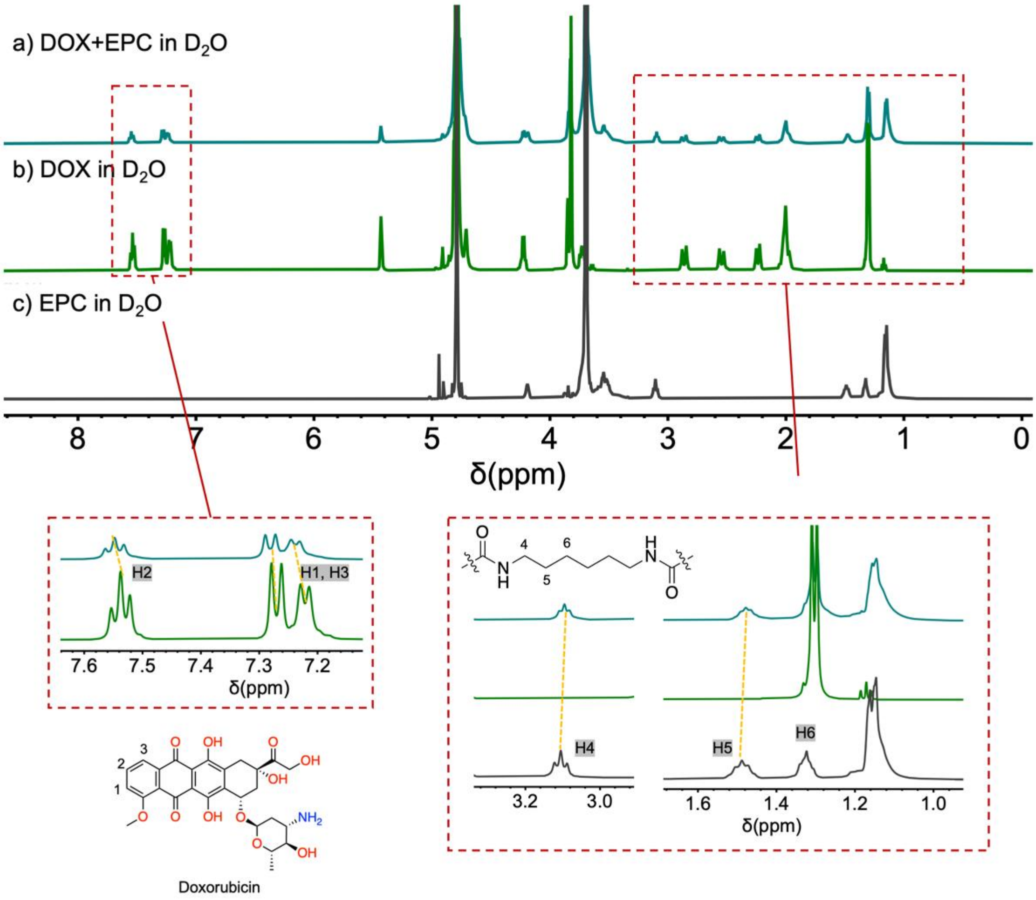

Figure S8: ^{1}H NMR spectra (in D_2O) of a) DOX loaded EPC, b) DOX and c) EPC. The inset shows the zoomed in region of the DOX aromatic protons and the urethane linkage in the EPC backbone.

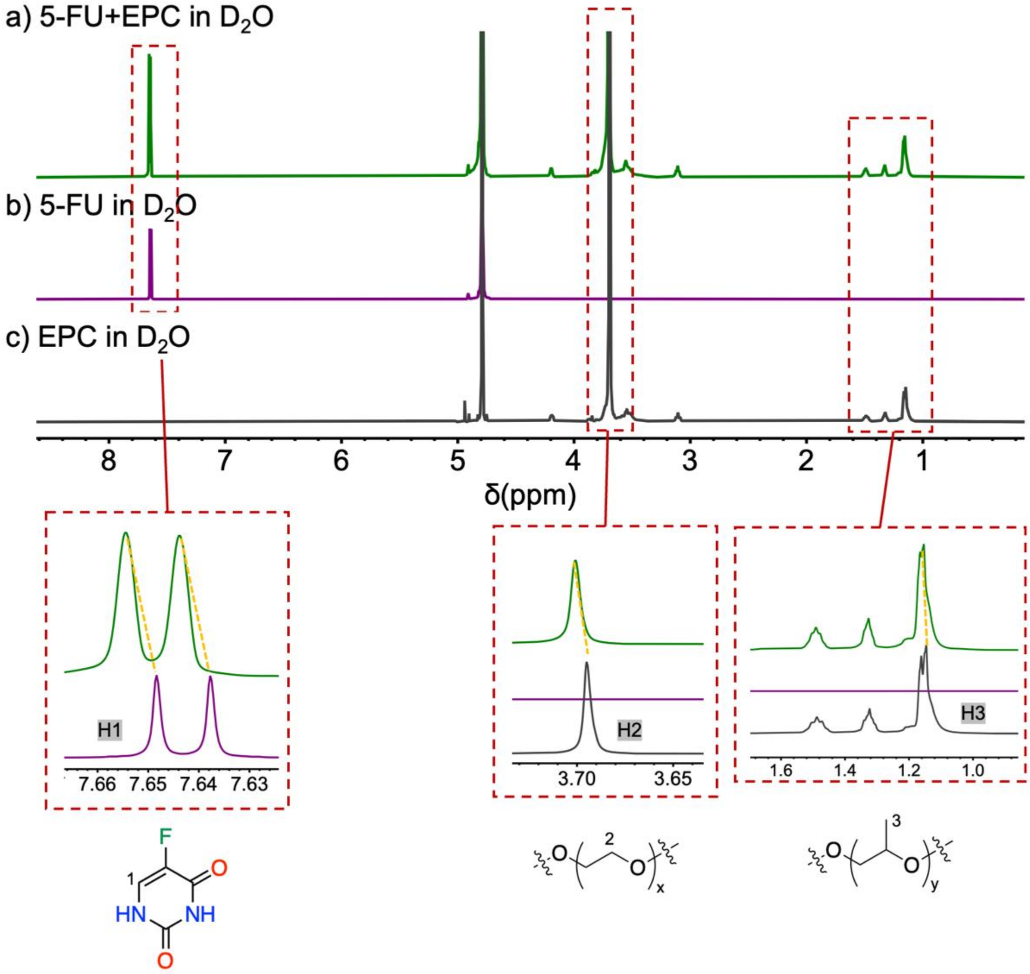

Figure S9: ^{1}H NMR spectra (in D_2O) of a) 5-FU loaded EPC, b) 5-FU and c) EPC. The inset shows the zoomed in region of the 5-FU protons and the protons from PEG and PPG segments in the EPC backbone.

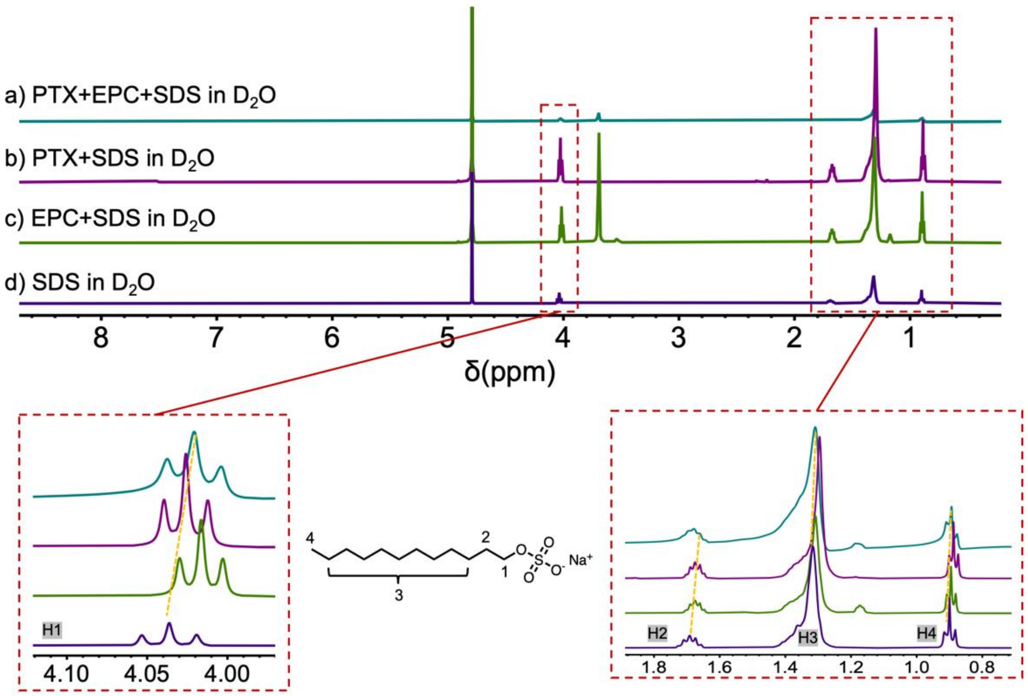

Figure S10: ^{1}H NMR spectra (in D_2O) of a) PTX and SDS loaded EPC, b) PTX and SDS, c) SDS loaded EPC and d) SDS. The inset shows the zoomed in region of the SDS protons. No obvious shifting was observed in the PTX protons and the protons from PEG and PPG segments were obscured due to overlaps with proton peaks from the SDS, preventing their characterization.

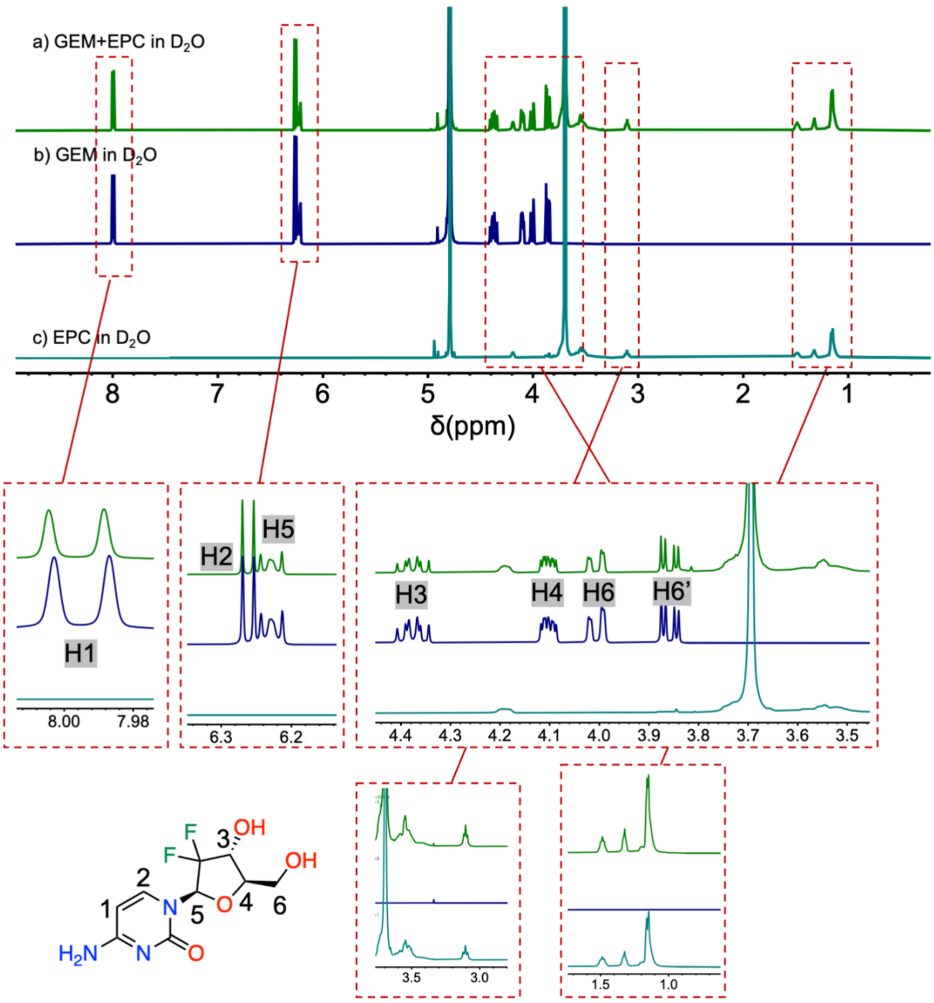

Figure S11: ^{1}H NMR spectra (in D_2O) of a) GEM loaded EPC, b) GEM and c) EPC. The inset shows the zoomed in region of the GEM protons and the protons from PEG and PPG segments in the EPC backbone. The comparison indicates no obvious peak shifting in GEM loaded EPC.

S7 Oscillatory temperature sweep rheological measurement

For preparing the drug@Gel composite, 15 wt% EPC gel was mixed with 100 μL drug solution (0.5 mg/mL) in an Eppendorf tube while the gel was in its cold, sol state. Then mixture was equilibrated at 4 °C with intermittent vortexing to ensure homogenous distribution of drug within the gel matrix. Afterwards the drug@Gel composite was allowed to thermogelate at 37 °C.

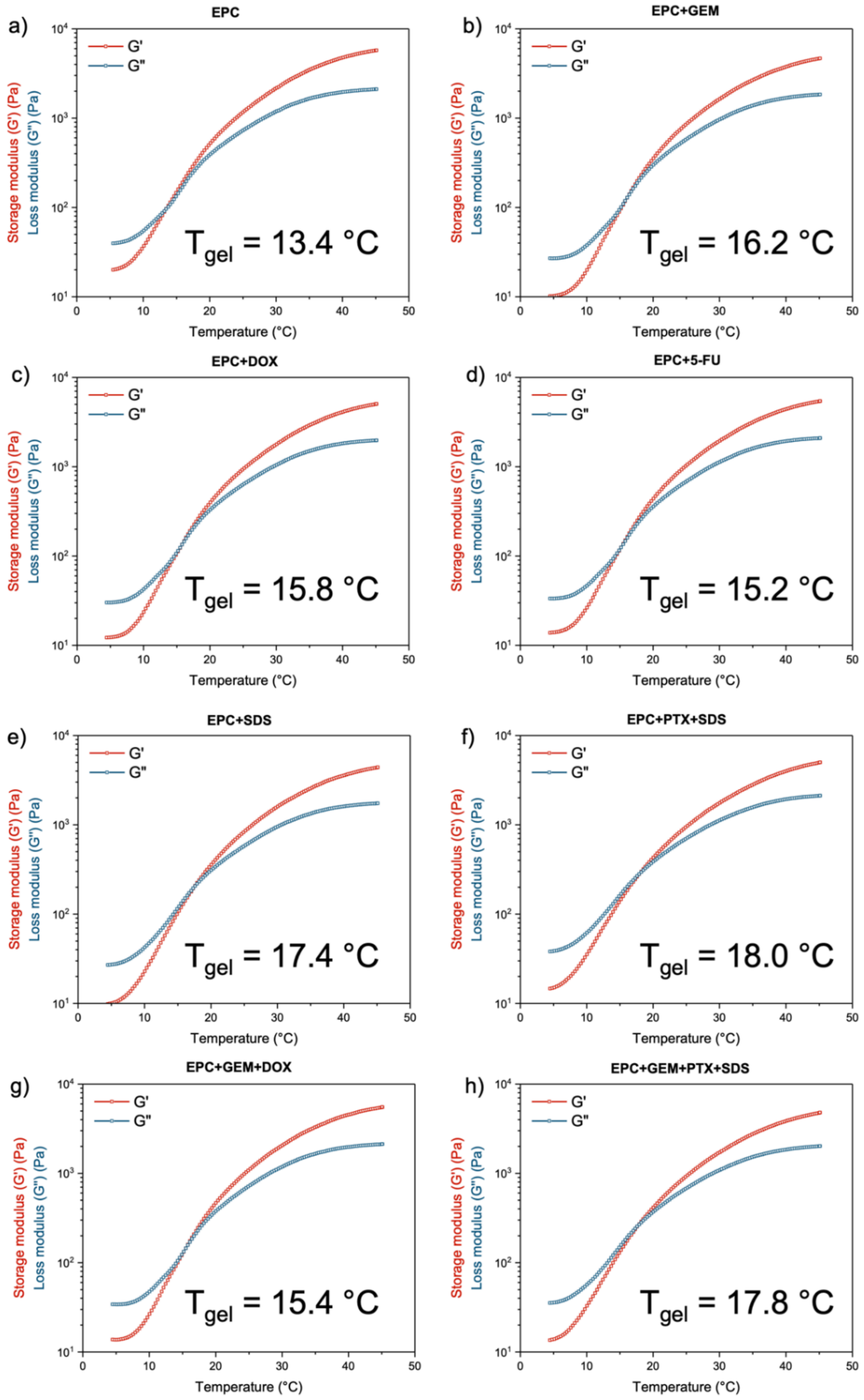

Figure S12: Temperature-dependent rheological properties of drug loaded EPC gel: a) EPC, b) EPC+GEM, c) EPC+DOX, d) EPC+5-FU, e) EPC+SDS, f) EPC+PTX+SDS, g) EPC+GEM+DOX and h) EPC+GEM+PTX+SDS.

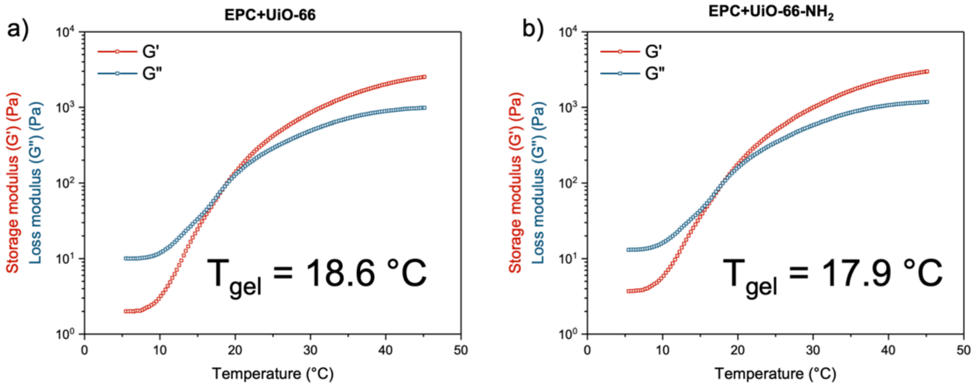

Figure S13: Temperature-dependent rheological properties of MOF loaded EPC gel: a) EPC loaded with UiO-66 and b) EPC loaded with UiO-66-NH_2

Table S1: Oscillatory temperature sweep measurements of a solution of EPC in deionized water (15 wt%) loaded with different drugs.

Sample[a]	T_{gel} (°C)	Storage Modulus (G') at 37°C (kPa)	Mesh size (nm) [b]
EPC (blank)	13.4	4.0	10.2
EPC (SDS 0.03 w/v%)	17.4	3.0	11.3
EPC+GEM	16.2	3.1	11.1
EPC+5-FU	15.2	3.7	10.5
EPC+DOX	15.8	3.4	10.8
EPC+PTX (SDS 0.03 w/v%)	18.0	3.3	10.9
EPC+DOX+GEM	15.4	3.8	10.4
EPC+GEM+PTX (SDS 0.03 w/v%)	17.8	3.2	11.0

[a] The EPC (SDS 0.03 w/v%) sample was prepared by dissolving 70 mg EPC in 400 μL DI H_2O with SDS (0.03 w/v%) under 4 °C. For the single-drug loaded EPC gel, 100 μL stock solution of drug (0.5 mg/mL in DI H_2O) was mixed with 70 mg EPC gel solution in 300 μL DI H_2O under 4 °C. For the dual-drug loaded EPC gel, 50 μL stock solution of each drug (1 mg/mL in DI H_2O) was mixed with 70 mg EPC gel solution in 300 μL DI H_2O under 4 °C. EPC+GEM+PTX (SDS 0.03 w/v%) was prepared by mixing 50 μL stock solution of GEM and PTX (1 mg/mL in DI H_2O, respectively) was mixed with 70 mg EPC gel solution in 300 μL DI H_2O with SDS (0.03 w/v%) under 4 °C
[b] Mesh size was determined from the storage modulus at 37 °C using rubber elastic theory. Mesh size (ξ) = $(N_A RT/G')^{1/3}$,[4] where N_A is the Avogadro's constant, R is the ideal gas constant, T is the absolute temperature in Kelvin and G' is the storage modulus.

Table S2: Oscillatory temperature sweep measurements of a solution of EPC in deionized water (15 wt%) loaded with MOFs (0.9 wt%).

Sample[a]	T_{gel} (°C)	Storage Modulus (G') at 37°C (kPa)	Mesh size (nm) [b]
EPC (blank)	13.4	4.0	10.2
UiO-66/EPC	18.6	1.7	13.7
UiO-66-NH_2/EPC	17.9	2.0	13.0

[a] The blank EPC sample was prepared by dissolving 70 mg EPC in 400 µL deionized water (DI H_2O) under 4 °C. For the MOF loaded EPC gel, 200 µL MOF stock solution (3 mg/mL in DI H_2O) was mixed with 70 mg EPC gel solution in 200 µL deionized water under 4 °C.

[b] Mesh size was determined from the storage modulus at 37 °C using rubber elastic theory. Mesh size (ξ) = $(N_A RT/G')^{1/3}$,[4] where N_A is the Avogadro's constant, R is the ideal gas constant, T is the absolute temperature in Kelvin and G' is the storage modulus.

S8 Additional drug release studies

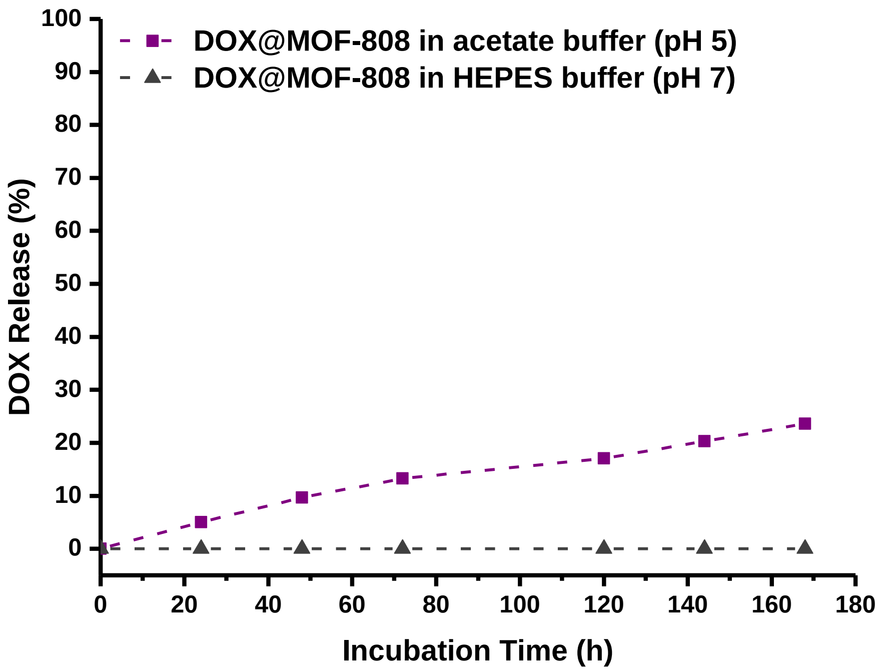

Figure S14: DOX release from DOX@MOF-808 in HEPES buffer (pH 7) and in acetate buffer (pH 5).

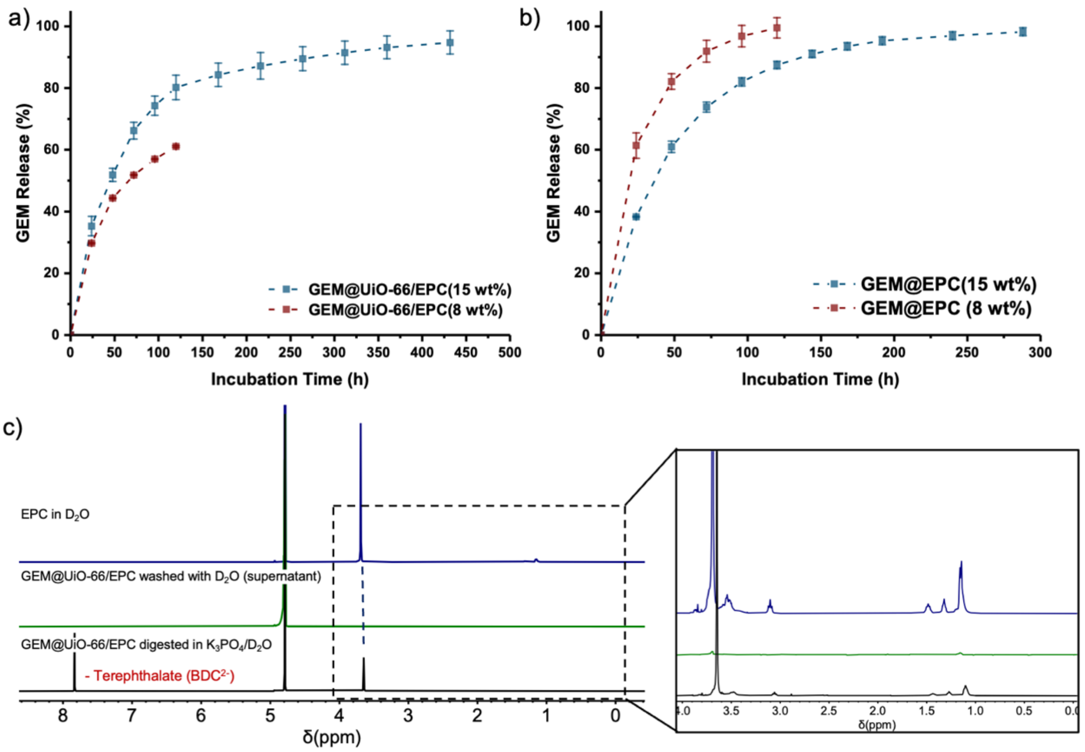

Figure S15: a) GEM release from GEM@UiO-66 in different EPC gel fraction (8 wt% and 15 wt%). b) GEM release from different EPC gel fraction (8 wt% and 15 wt%). c) ^{1}H-NMR of digested UiO-66 from drug release experiment, indicating the uptake of EPC gel.

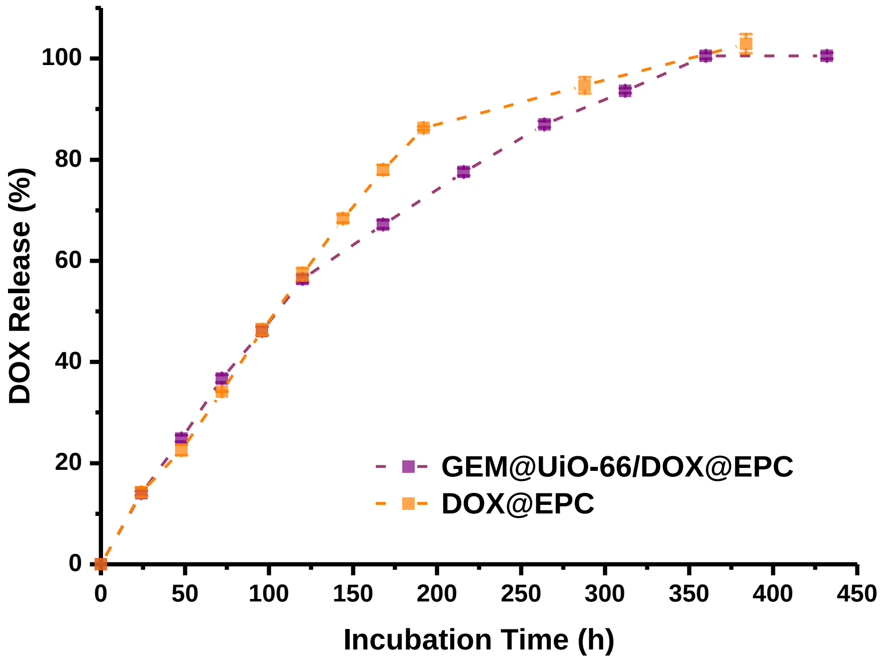

Figure S16: a) Comparative DOX release in DOX@EPC (15 wt%) and GEM@UiO-66/DOX@EPC (15 wt%).

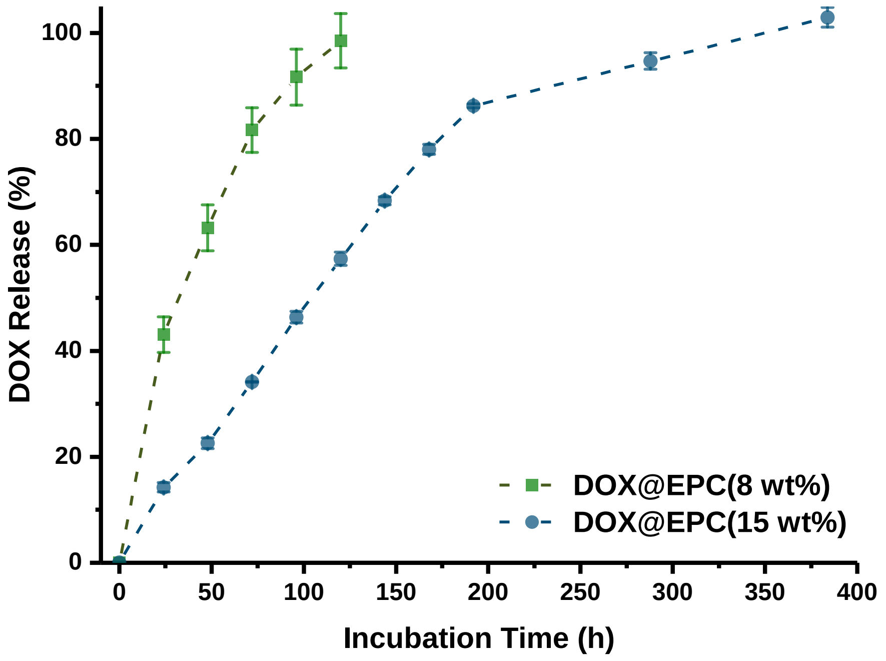

Figure S17: Cumulative release profiles of DOX from DOX@EPC with different EPC gel fractions (8 wt% and 15 wt%) at 37 °C. Data were fitted with the Korsmeyer–Peppas model, yielding a release exponent of n = 0.52 (8 wt%) and n = 0.95 (15 wt%).

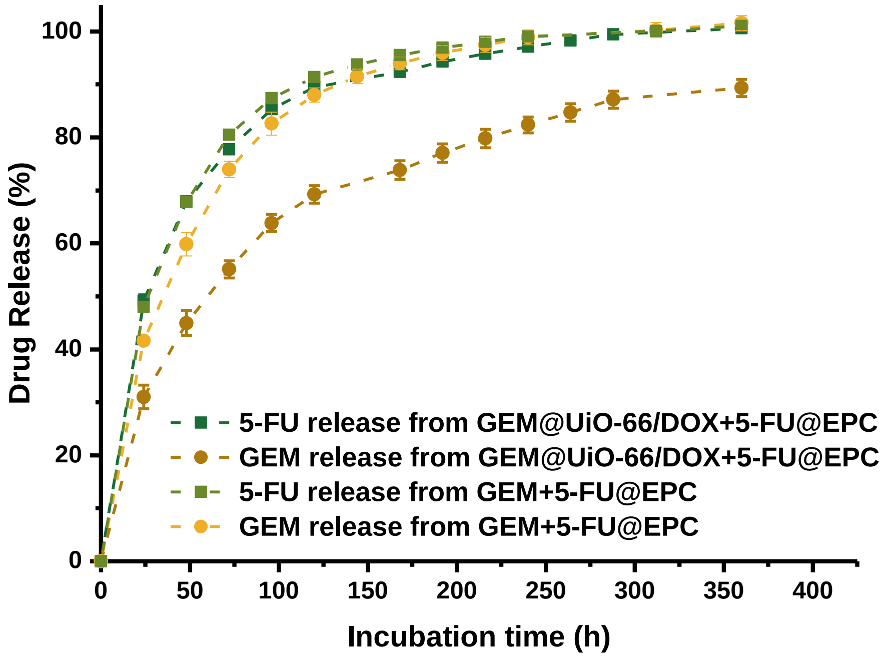

Figure S18: Comparison of the 5-FU and GEM release from GEM@UiO-66/DOX+5-FU@EPC and GEM+5-FU@EPC, showing that the encapsulation of GEM within UiO-66 can effectively slow down the GEM release.

S9 Calibration curve of GEM, DOX, 5-FU

To quantify the amount of drug released, calibration curves for gemcitabine (GEM), doxorubicin (DOX) and 5-fluorouracil (5-FU) were established in 25 mM HEPES buffer (pH 7). Each drug was analysed at various concentrations, and the resulting curves exhibited good linearity based on linear regression analysis.

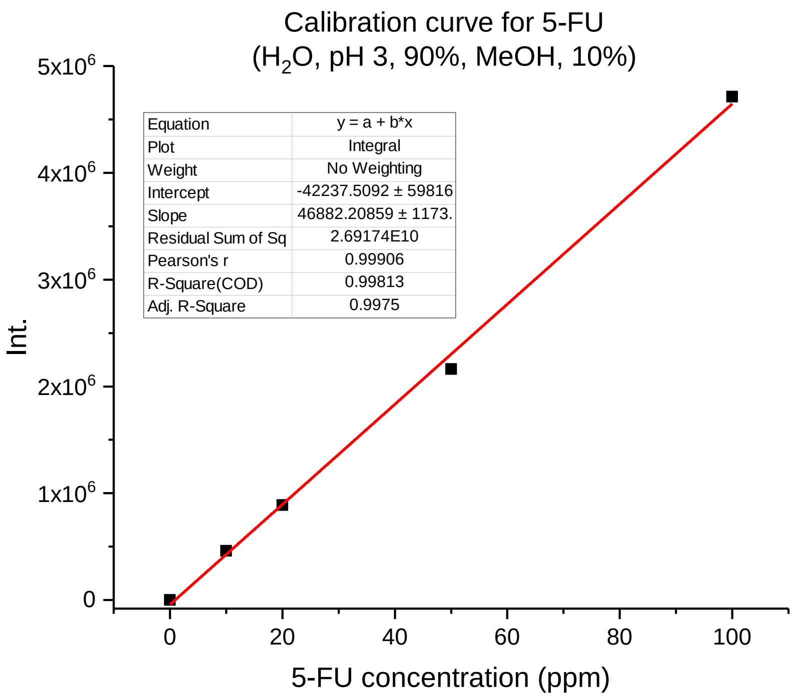

Figure S19: Calibration curve of 5-FU.

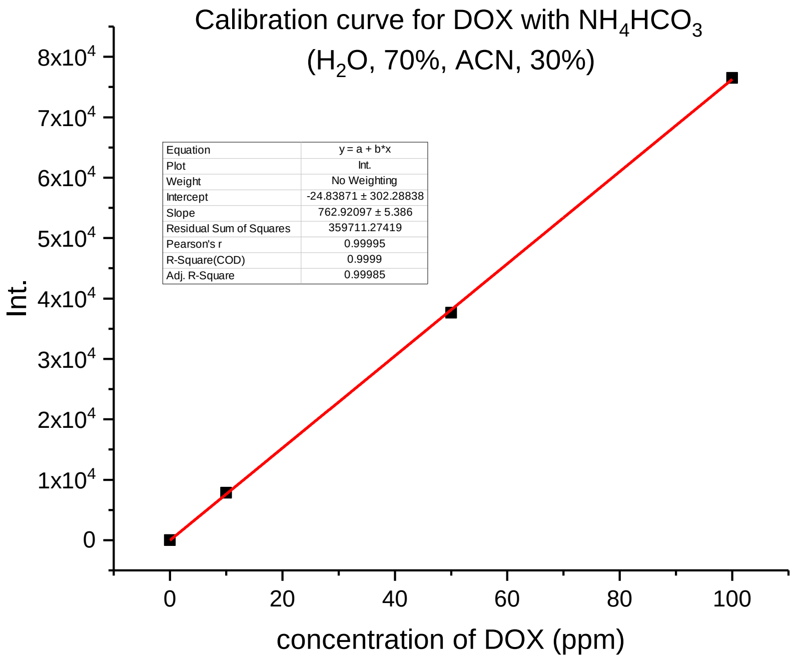

Figure S20: Calibration curve of DOX in the presence of NH_4HCO_3.

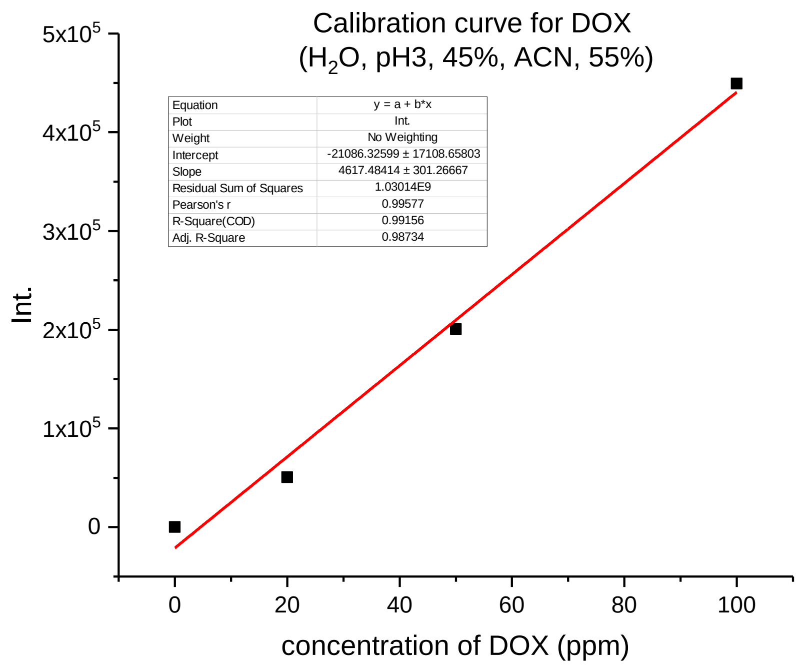

Figure S21: Calibration curve of DOX.

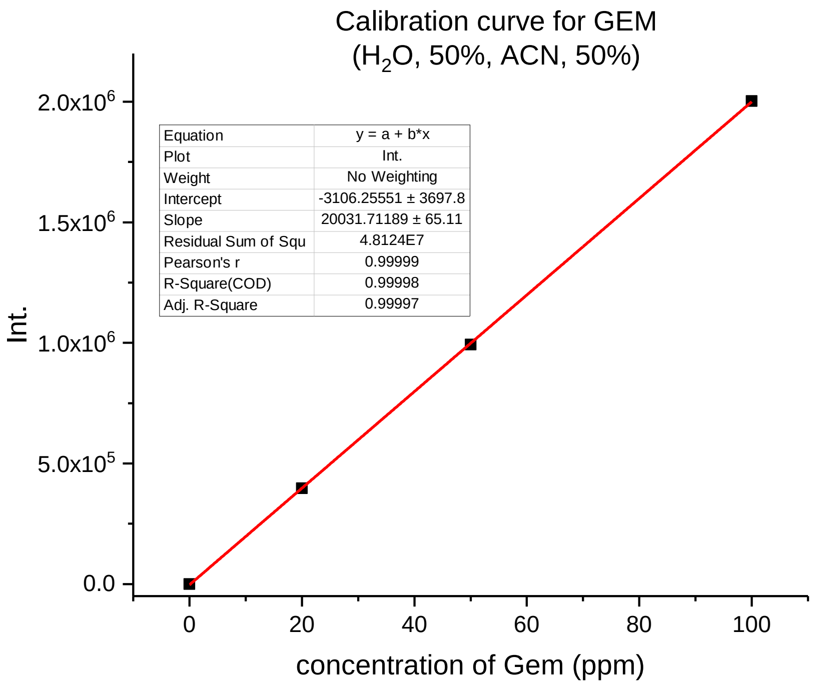

Figure S22: Calibration curve of GEM.

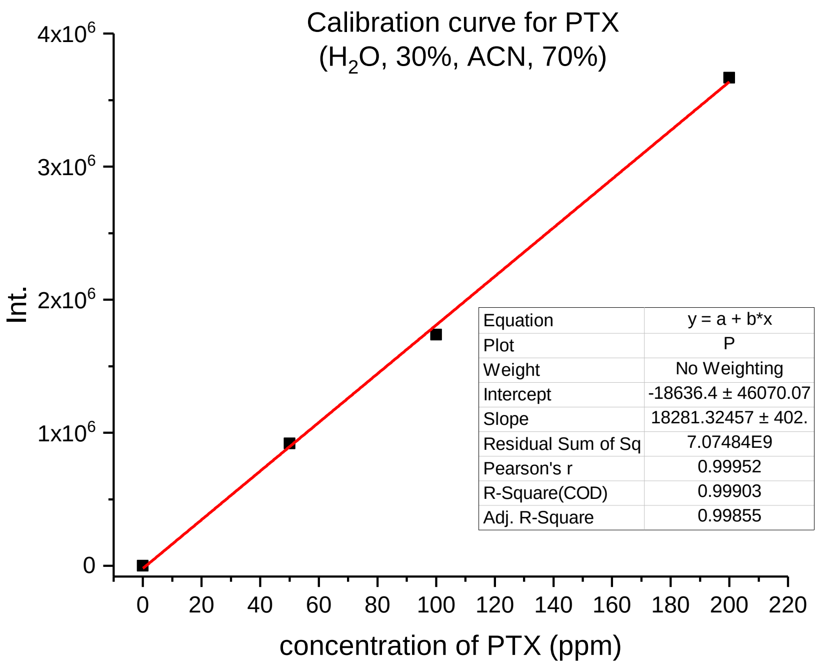

Figure S23: Calibration curve of PTX.

S10 Korsmeyer-Peppas fitting for the release kinetic

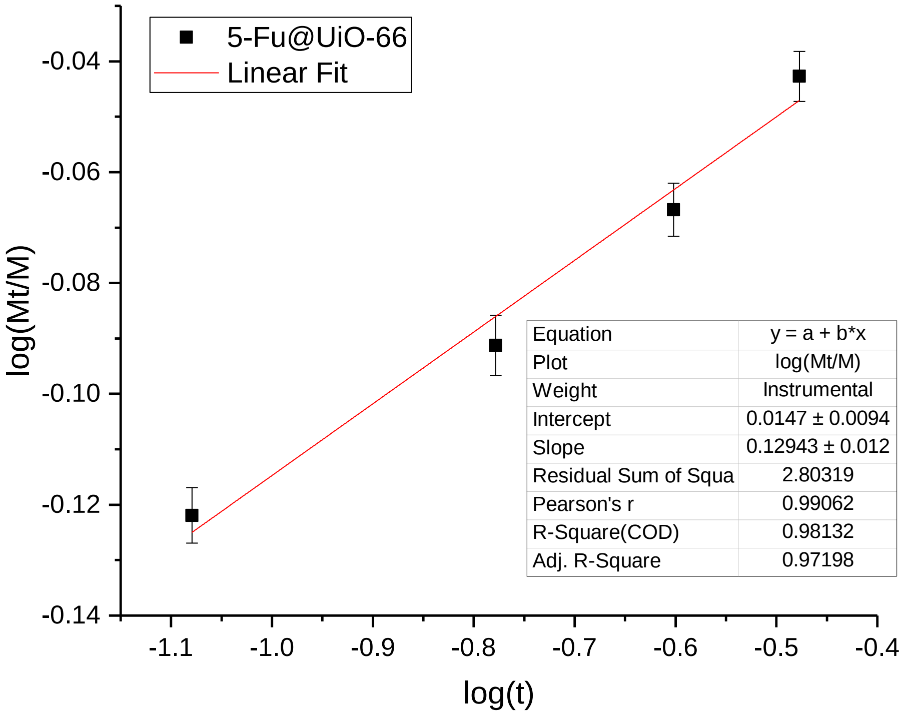

Figure S24: Korsmeyer-Peppas Fitting for the 5-FU release from 5-FU@UiO-66 in HEPES Buffer (25 mM, pH 7).

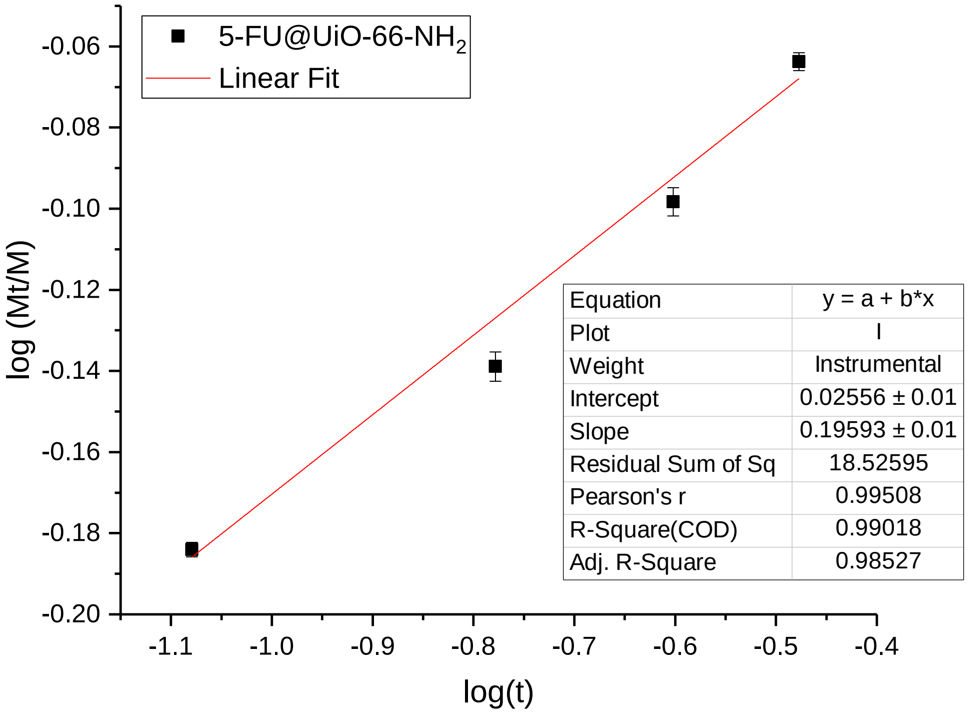

Figure S25: Korsmeyer-Peppas Fitting for the 5-FU release from 5-FU@UiO-66-NH_2 in HEPES Buffer (25 mM, pH 7).

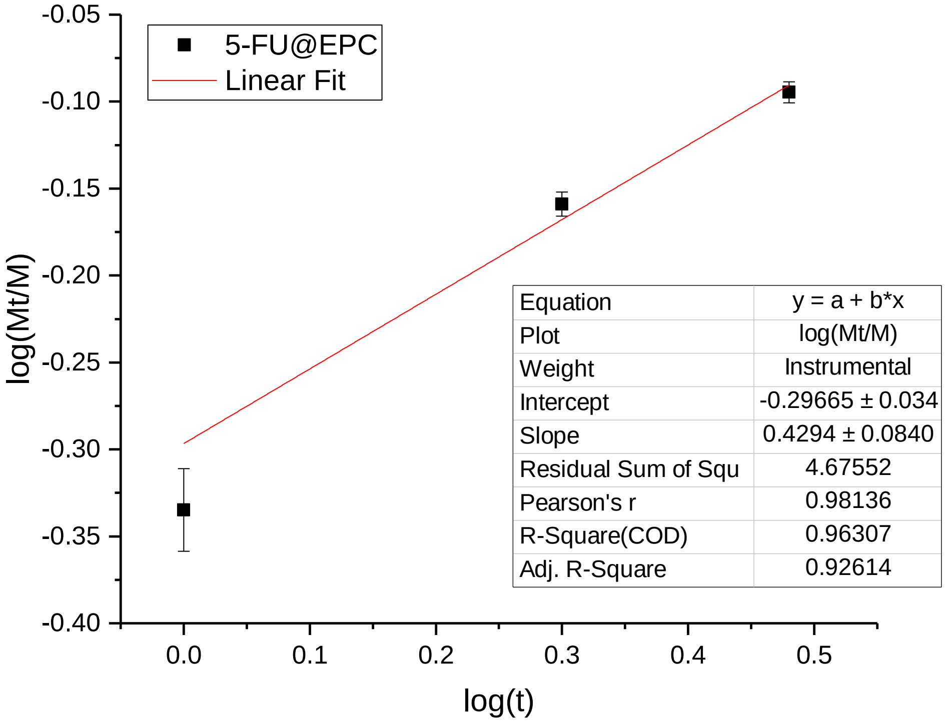

Figure S26: Korsmeyer-Peppas Fitting for the 5-FU release from 5-FU@EPC in HEPES Buffer (25 mM, pH 7).

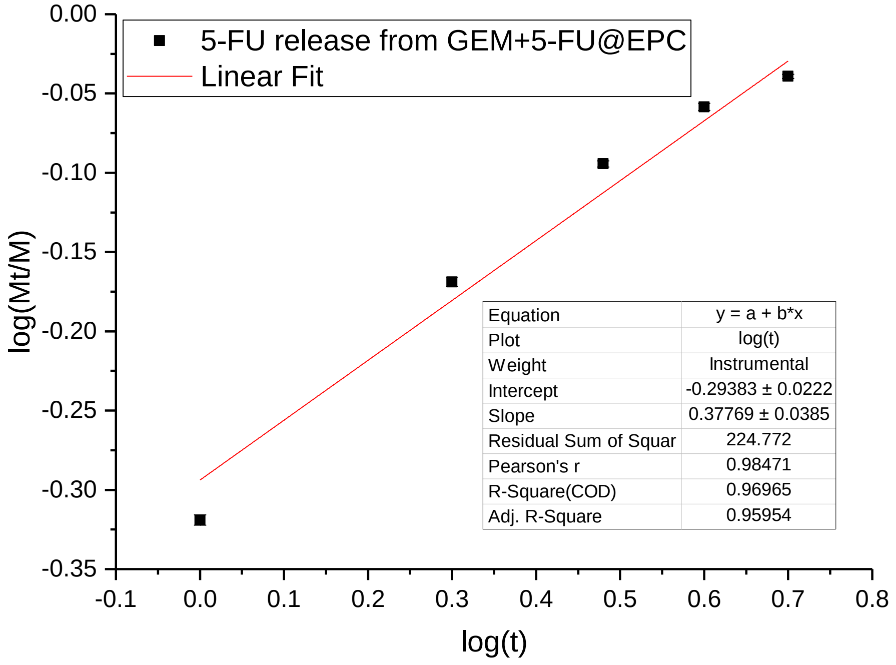

Figure S27: Korsmeyer-Peppas Fitting for the 5-FU release from GEM+5-FU@EPC in HEPES Buffer (25 mM, pH 7).

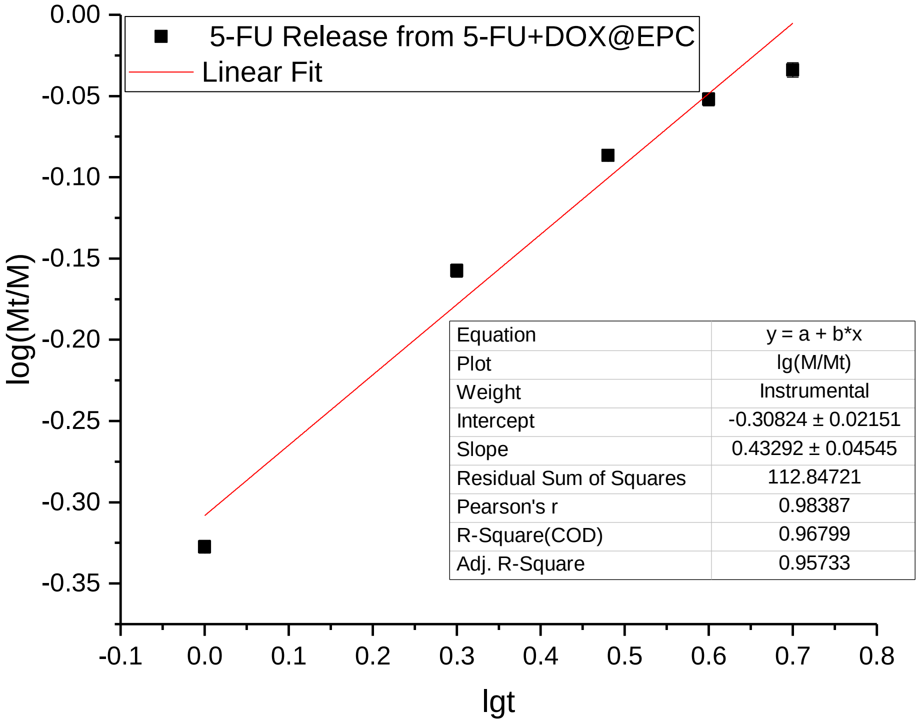

Figure S28: Korsmeyer-Peppas Fitting for the 5-FU release from 5-FU+DOX@EPC in HEPES Buffer (25 mM, pH 7).

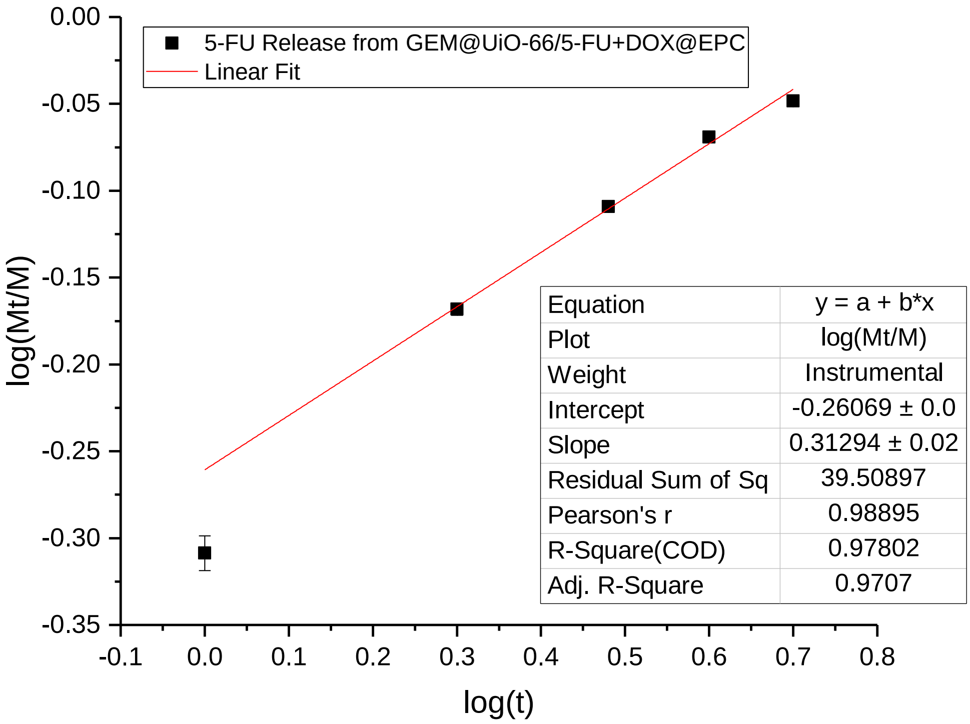

Figure S29: Korsmeyer-Peppas Fitting for the 5-FU release from GEM@UiO-66/5-FU+DOX@EPC in HEPES Buffer (25 mM, pH 7).

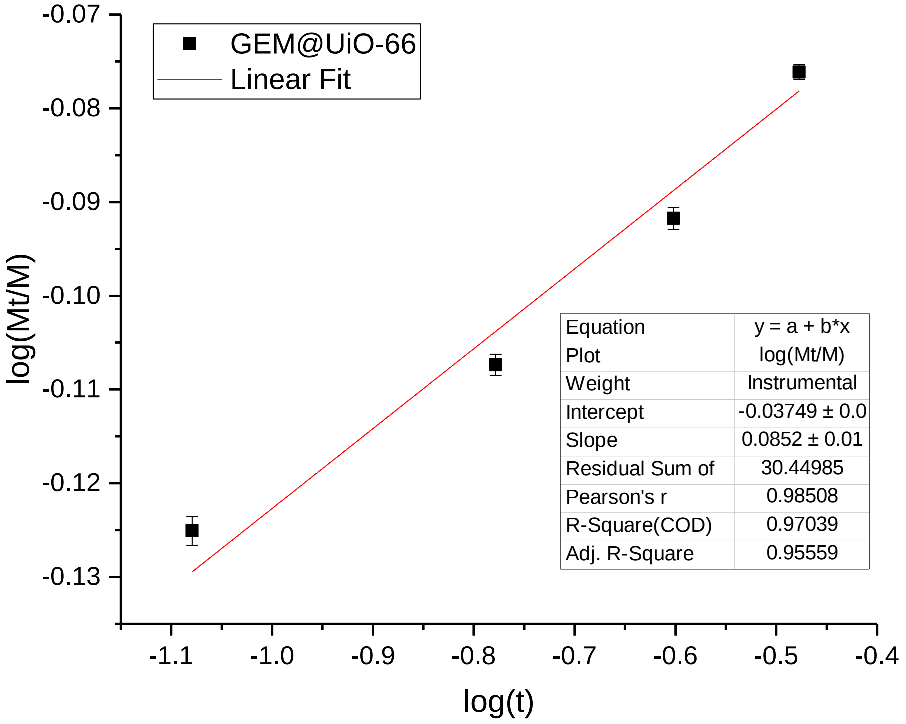

Figure S30: Korsmeyer-Peppas Fitting for the GEM release from GEM@UiO-66 in HEPES Buffer (25 mM, pH 7).

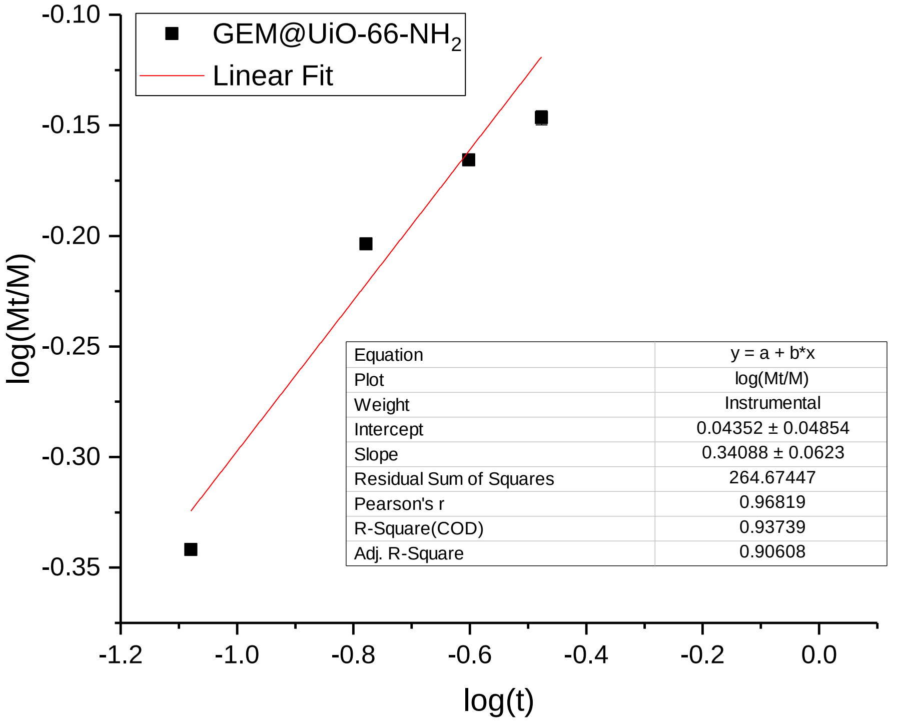

Figure S31: Korsmeyer-Peppas Fitting for the GEM release from GEM@UiO-66-NH_2 in HEPES Buffer (25 mM, pH 7).

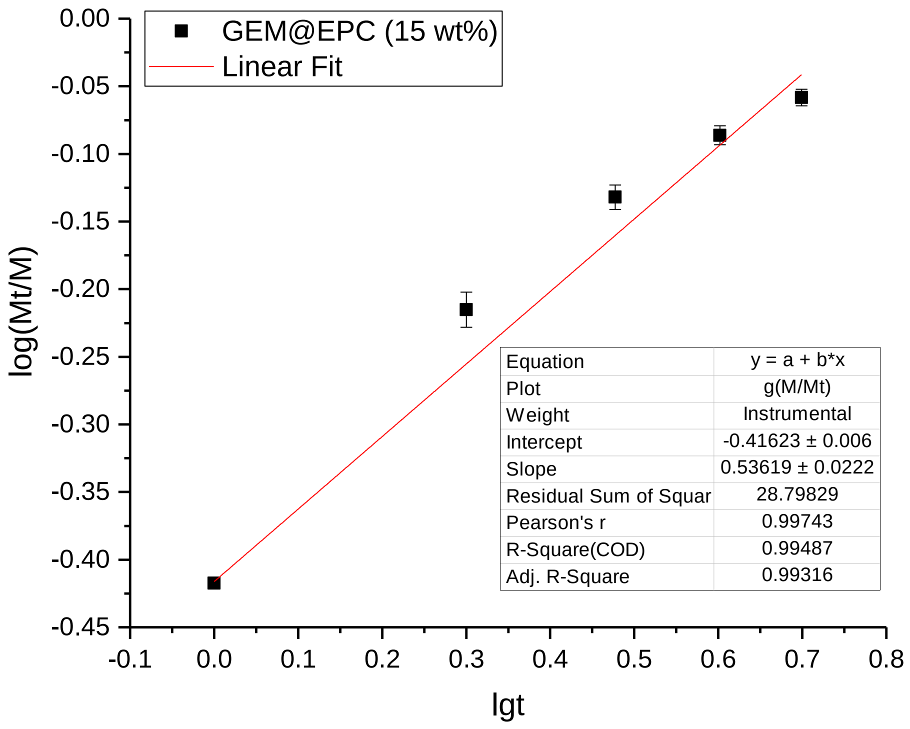

Figure S32: Korsmeyer-Peppas Fitting for the GEM release from GEM@EPC (15 wt%) in HEPES Buffer (25 mM, pH 7).

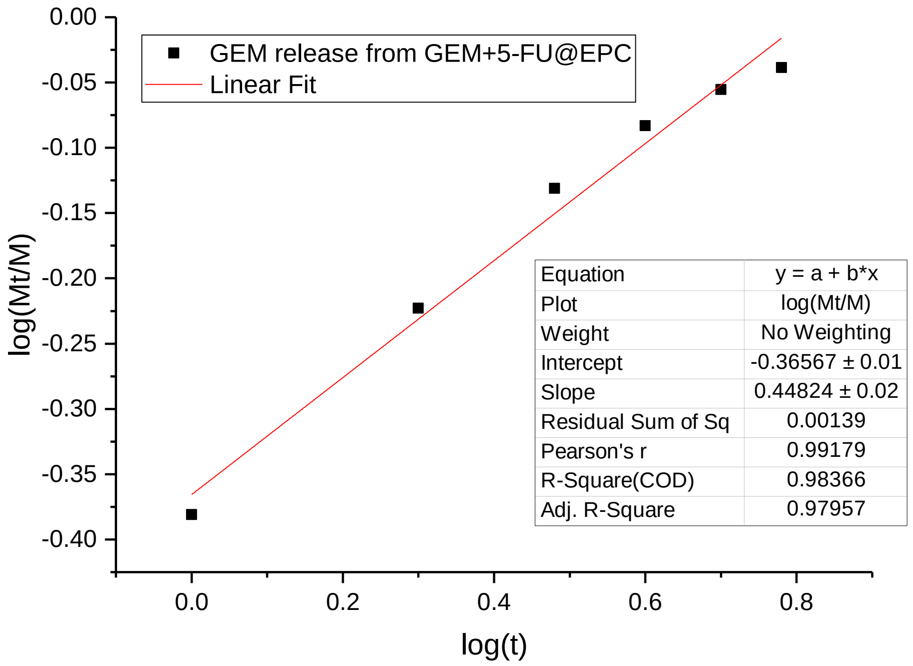

Figure S33: Korsmeyer-Peppas Fitting for the GEM release from GEM+5-FU@EPC in HEPES Buffer (25 mM, pH 7).

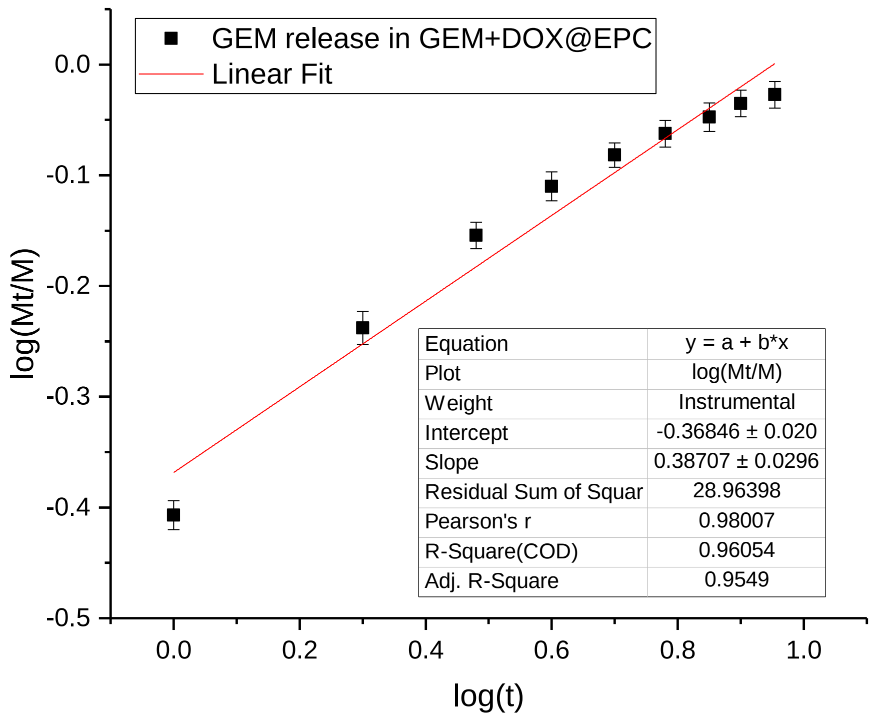

Figure S34: Korsmeyer-Peppas Fitting for the GEM release from GEM+DOX@EPC in HEPES Buffer (25 mM, pH 7).

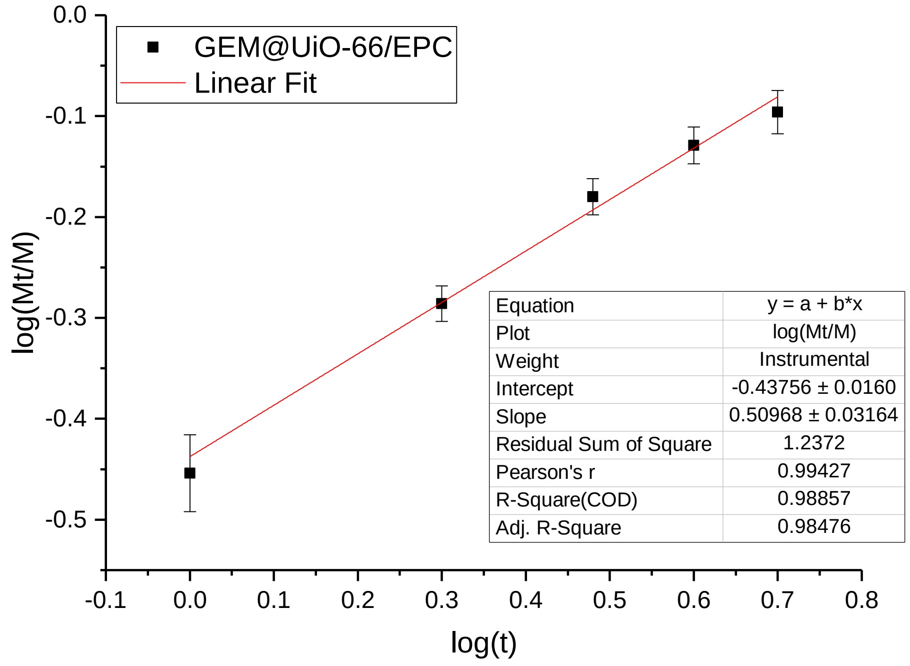

Figure S35: Korsmeyer-Peppas Fitting for the GEM release from GEM@UiO-66/EPC in HEPES Buffer (25 mM, pH 7).

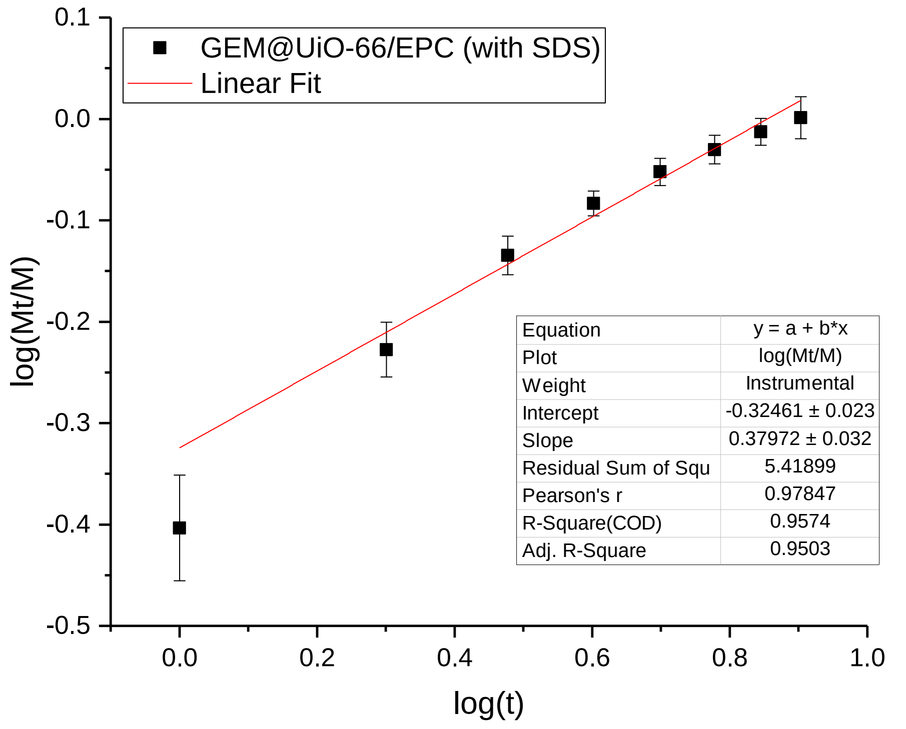

Figure S36: Korsmeyer-Peppas Fitting for the GEM release from GEM@UiO-66/EPC in HEPES Buffer (25 mM, pH 7, with 0.03 w/v% SDS).

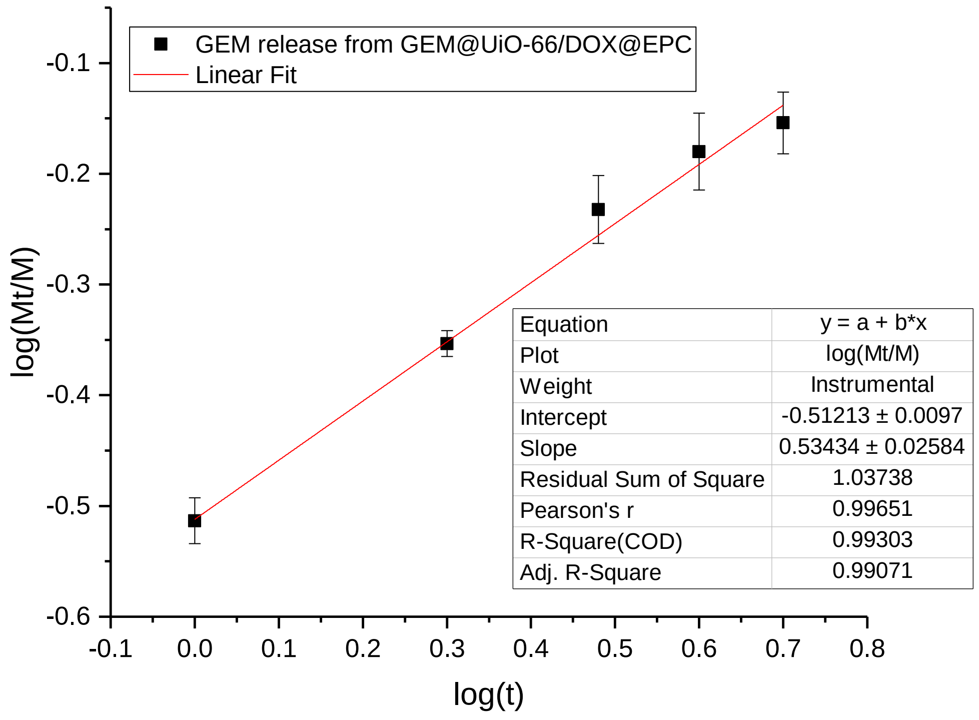

Figure S37: Korsmeyer-Peppas Fitting for the GEM release from GEM@UiO-66/DOX@EPC in HEPES Buffer (25 mM, pH 7).

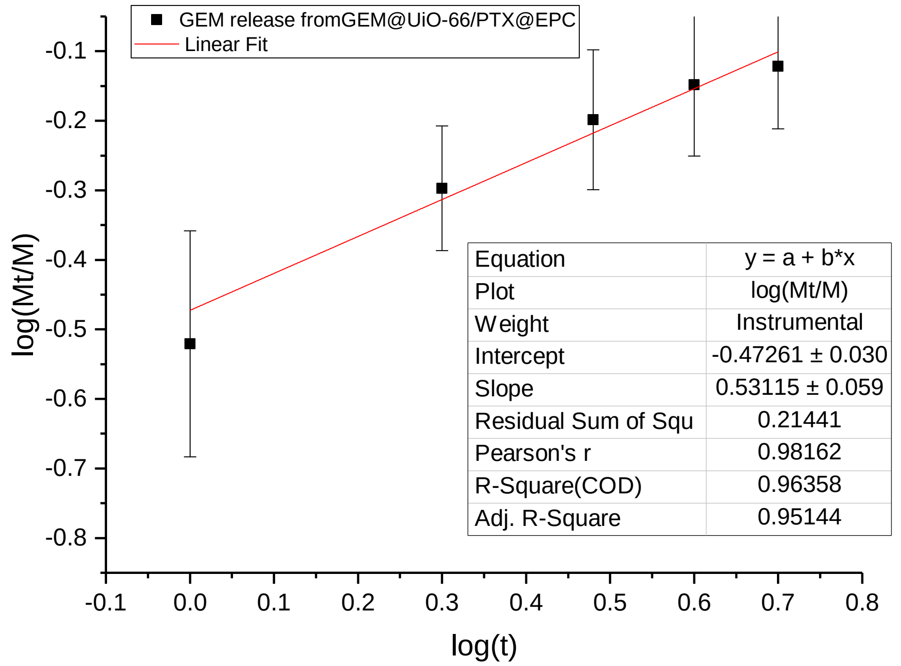

Figure S38: Korsmeyer-Peppas Fitting for the GEM release from GEM@UiO-66/PTX@EPC in HEPES Buffer (25 mM, pH 7, with 0.03 w/v% SDS).

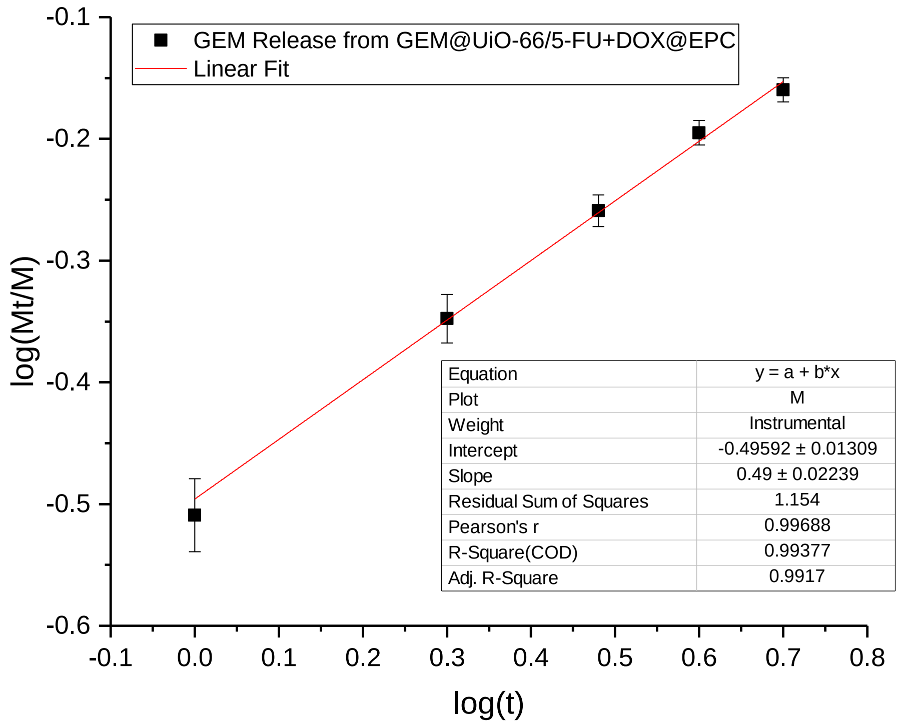

Figure S39: Korsmeyer-Peppas Fitting for the GEM release from GEM@UiO-66/5-FU+DOX@EPC in HEPES Buffer (25 mM, pH 7).

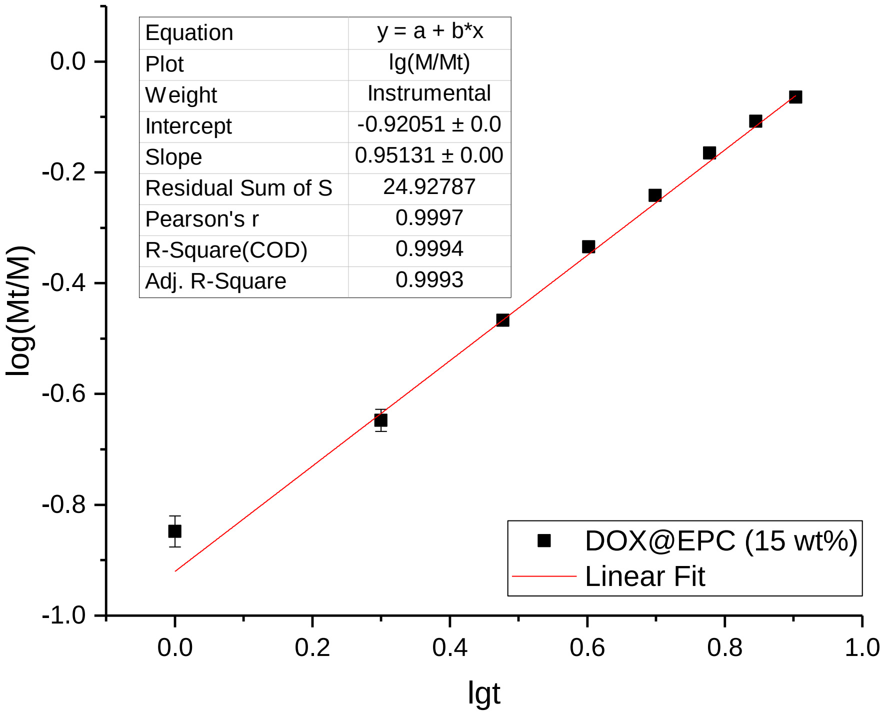

Figure S40: Korsmeyer-Peppas Fitting for the DOX release from DOX@EPC (15 wt%) in HEPES Buffer (25 mM, pH 7).

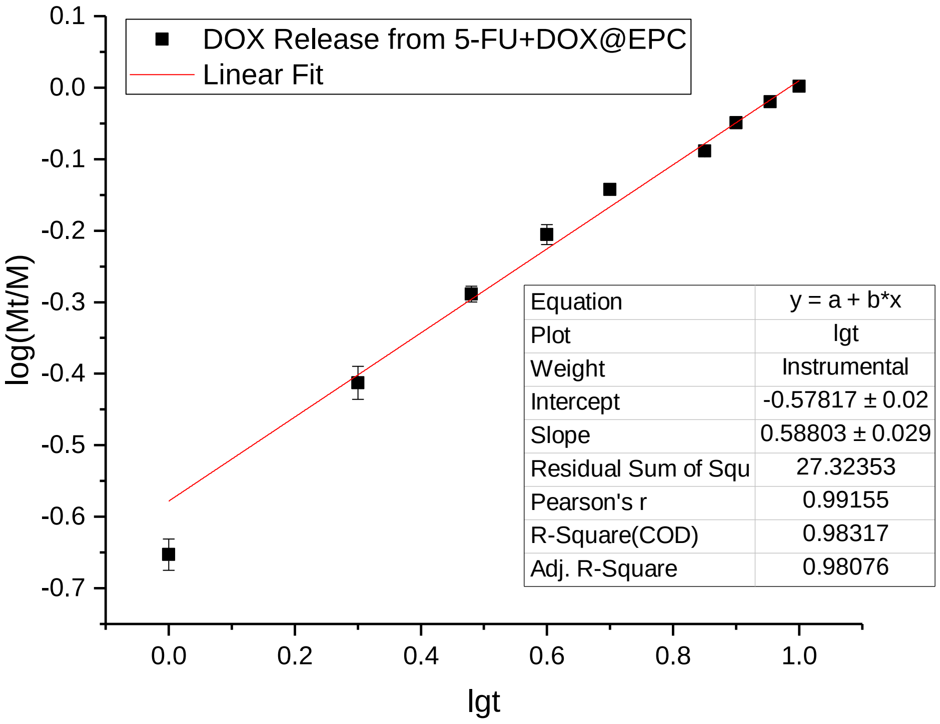

Figure S41: Korsmeyer-Peppas Fitting for the DOX release from 5-FU+DOX@EPC in HEPES Buffer (25 mM, pH 7).

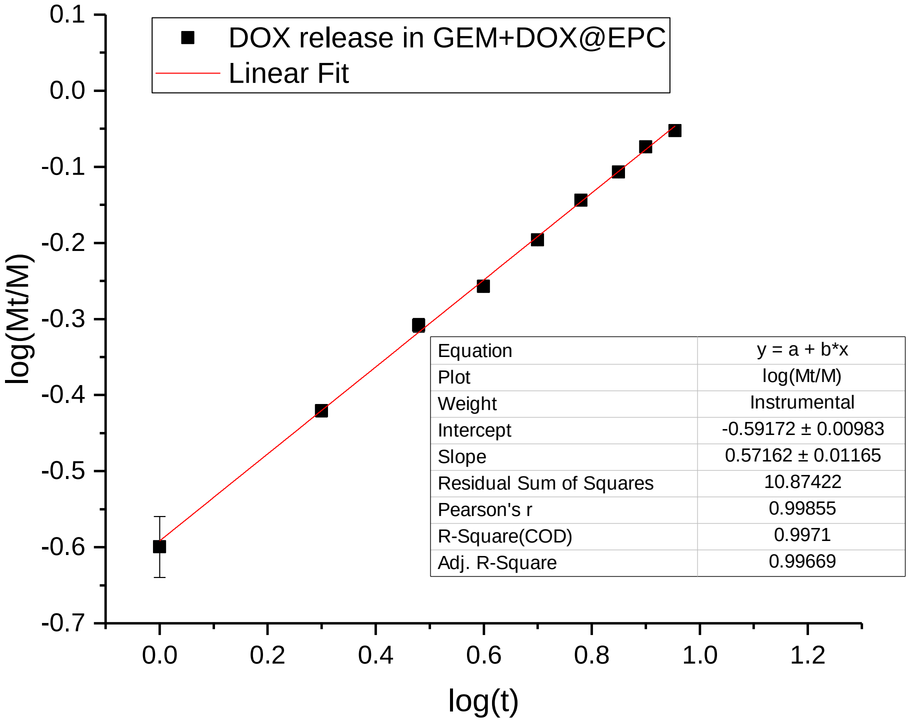

Figure S42: Korsmeyer-Peppas Fitting for the DOX release from GEM+DOX@EPC in HEPES Buffer (25 mM, pH 7).

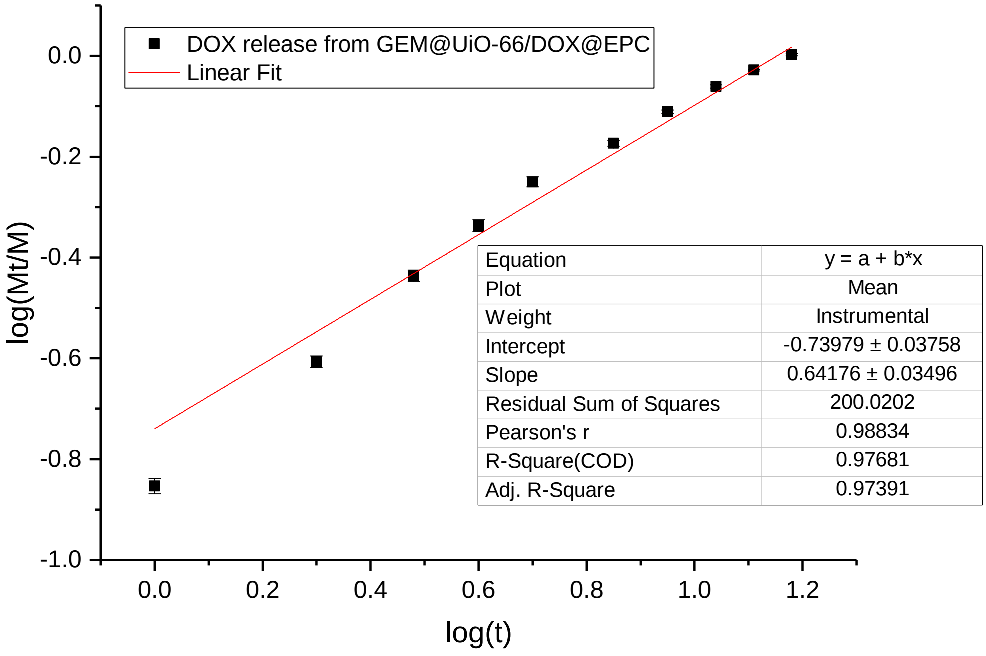

Figure S43: Korsmeyer-Peppas Fitting for the DOX release from GEM@UiO-66/DOX@EPC in HEPES Buffer (25 mM, pH 7).

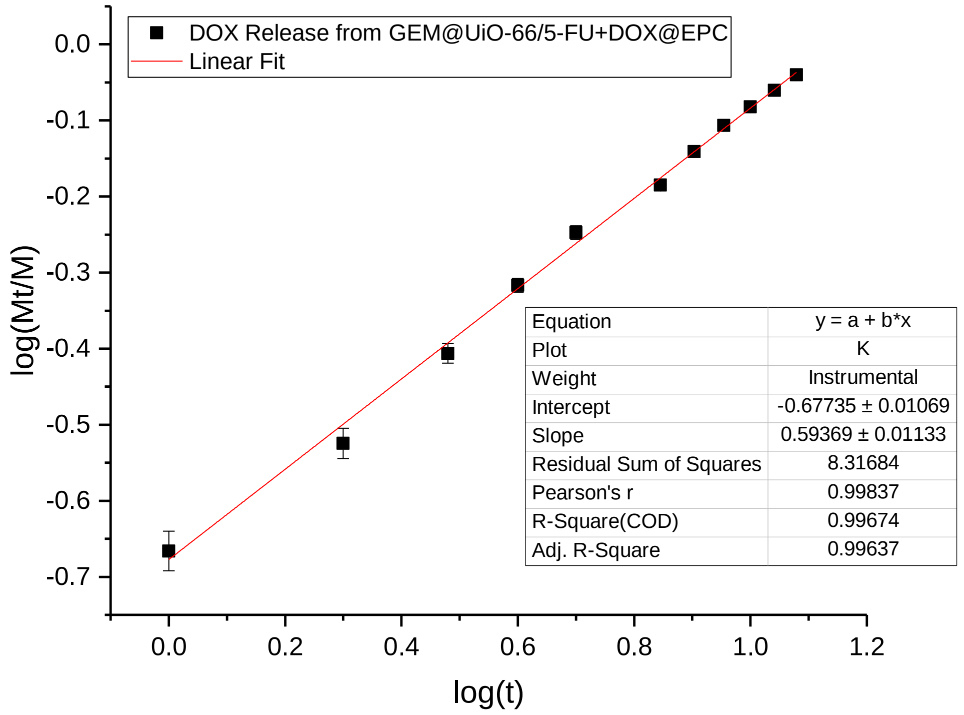

Figure S44: Korsmeyer-Peppas Fitting for the DOX release from GEM@UiO-66/5-FU+DOX@EPC in HEPES Buffer (25 mM, pH 7).

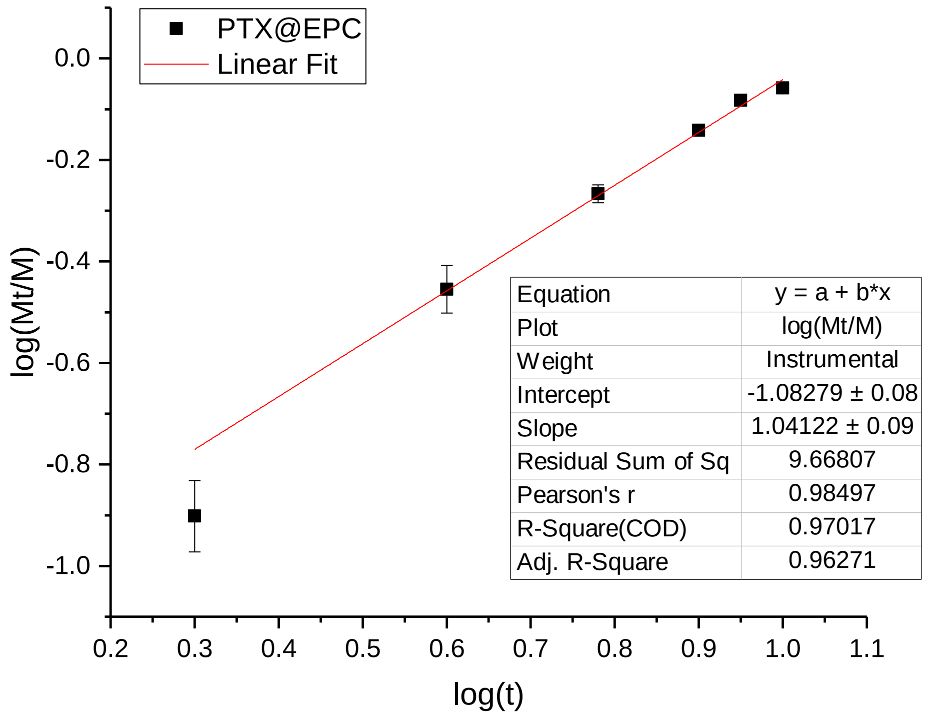

Figure S45: Korsmeyer-Peppas Fitting for the PTX release from PTX@EPC in HEPES Buffer (25 mM, pH 7, with 0.03 w/v% SDS).

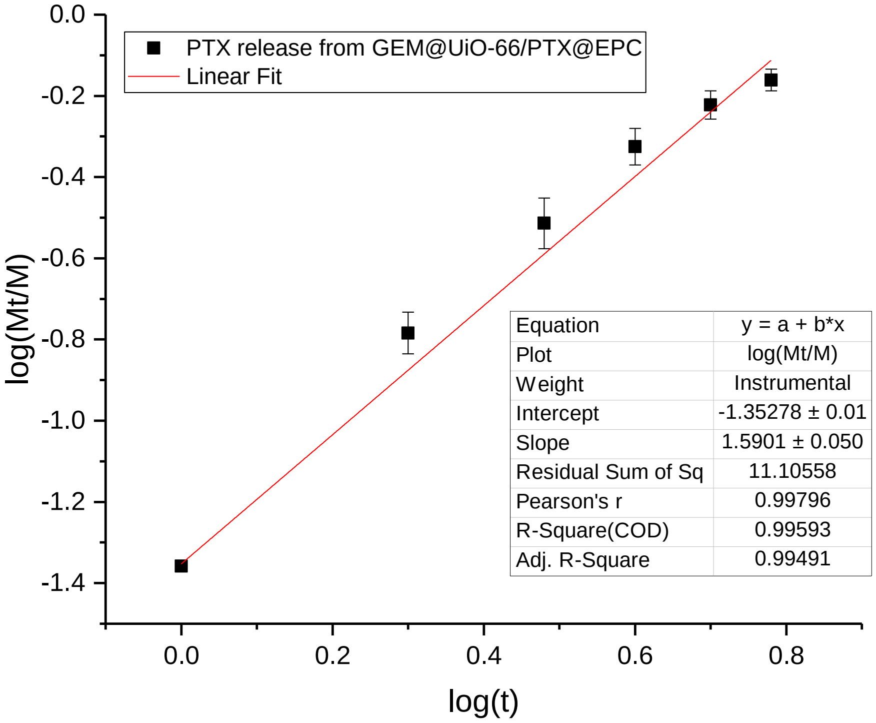

Figure S46: Korsmeyer-Peppas Fitting for the PTX release from GEM@UiO-66/PTX@EPC in HEPES Buffer (25 mM, pH 7, with 0.03 w/v% SDS).

Reference

1. Cavka, J. H.; Jakobsen, S.; Olsbye, U.; Guillou, N.; Lamberti, C.; Bordiga, S.; Lillerud, K. P., A New Zirconium Inorganic Building Brick Forming Metal Organic Frameworks with Exceptional Stability. *J. Am. Chem. Soc.* **2008,** *130* (42), 13850-13851.
2. Furukawa, H.; Gándara, F.; Zhang, Y.-B.; Jiang, J.; Queen, W. L.; Hudson, M. R.; Yaghi, O. M., Water Adsorption in Porous Metal–Organic Frameworks and Related Materials. *J. Am. Chem. Soc.* **2014,** *136* (11), 4369-4381.
3. Morris, W.; Stevens, C.; Taylor, R.; Dybowski, C.; Yaghi, O.; Garcia-Garibay, M., NMR and X-ray Study Revealing the Rigidity of Zeolitic Imidazolate Frameworks. *J. Phys. Chem. C* **2012,** *116*, 13307–13312.
4. Tsuji, Y.; Li, X.; Shibayama, M., Evaluation of Mesh Size in Model Polymer Networks Consisting of Tetra-Arm and Linear Poly(ethylene glycol)s. *Gels* **2018,** *4* (2), 50.
